# Diphosphine-protected ultrasmall gold nanoclusters: opened icosahedral Au_13_ and heart-shaped Au_8_ clusters†
†Electronic supplementary information (ESI) available: Detailed synthesis procedure, computational details, IR, ^31^P NMR, ESI-MS, details of the data collection and structure refinements, and detailed analysis of the computed spectra. Additional figures (Fig. S1–S5 and Scheme S1) and tables (Tables S1–S4). CCDC 1562504, 1562505 and 1577669 for **SD/Au1**, **SD/Au2**, and **SD/Au3**. CCDC 1577877–1577881 for **SD/Au2** at 93, 183, 243, 273, and 293 K. For ESI and crystallographic data in CIF or other electronic format see DOI: 10.1039/c7sc03566g


**DOI:** 10.1039/c7sc03566g

**Published:** 2017-12-04

**Authors:** Shan-Shan Zhang, Lei Feng, Ravithree D. Senanayake, Christine M. Aikens, Xing-Po Wang, Quan-Qin Zhao, Chen-Ho Tung, Di Sun

**Affiliations:** a Key Lab of Colloid and Interface Chemistry , Ministry of Education , School of Chemistry and Chemical Engineering , Shandong University , Jinan , 250100 , P. R. China . Email: dsun@sdu.edu.cn; b Department of Chemistry , Kansas State University , Manhattan , Kansas 66506 , USA

## Abstract

Due to distinctive quantum confinement effects, ultrasmall gold nanoparticles usually exhibit interesting electronic structure and molecular-like properties. However, the lack of atomically-precise structural information makes the understanding of them almost impossible, such as understanding the relationships between their compositions and unique properties. Herein, by reducing a diphosphine Au^I^ precursor (Au_2_(dppm)_2_Cl_2_; dppm = Ph_2_PCH_2_PPh_2_) with or without a S^2–^ releasing reagent, we enriched our knowledge of the members in the families of Au_13_ and Au_8_ by the structural determinations of two new dppm-protected gold nanoclusters, [Au_13_(dppm)_6_]^5+^ (**SD/Au1**) and [Au_8_(dppm)_4_S_2_]^2+^ (**SD/Au2**), respectively. Within **SD/Au1**, the Au_13_ kernel significantly deviates from the ideal *I*_h_ icosahedron by the elongation of three surface Au–Au bonds to ∼3.5 Å, giving it *C*_3_ symmetry, whereas **SD/Au2** has a novel heart-shaped *C*_2_ symmetric Au_8_S_2_ core (central Au_4_ tetrahedron + two Au_2_S units) protected by four μ_2_-dppm ligands in the outer shell. Of note, **SD/Au1** represents a rare Au_13_ nanocluster with an opened icosahedral geometry, and **SD/Au2** shows a new edge-shared “core + 4*exo*” structure type that has never been observed before. The electronic structures and optical absorption spectra of these systems are correlated with time-dependent density functional theory (TDDFT) calculations. Based on the spherical jellium model, the stability of the Au_13_ and Au_8_ nanoclusters can be ascribed to 8- and 2-electron superatoms with 1S^2^1P^6^ and 1S^2^ configurations, respectively. Interestingly, the cluster **SD/Au2** exhibits bright yellow luminescence with an emission maximum at 591 nm that slightly hypsochromically shifts to 581 nm upon cooling to 93 K. Our findings not only enrich the family of diphosphine-protected ultrasmall gold nanoclusters, but also demonstrate the rich variations of gold kernels during the transformation from a simple Au^I^ precursor to Au nanoclusters.

## Abstract 2

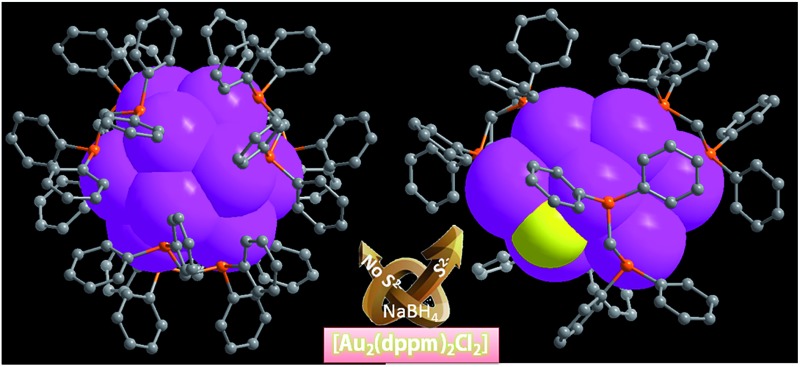
Two ultrasmall gold clusters, Au_13_ and Au_8_, were identified as a distorted *I*_h_ icosahedral Au_13_ and edge-shared “core + 4*exo*” structure Au_8_S_2_ cores, respectively. They showed interesting luminescence and electrochemical properties.

## Introduction

Gold nanoclusters with defined nuclearity and configurations have attracted considerable interest due to their nuclearity-selective optical, electronic, and catalytic properties which diverge significantly from those of their bulk metal counterparts.^1^ The total structure characterization of gold nanoclusters by X-ray crystallography is a prerequisite to the better understanding of their stability, metal–ligand interfacial bonding, as well as the aforementioned properties.^2^ Until now, phosphine,^3^ thiolate,^4^ and alkynyl-protected^5^ gold nanoclusters have been well identified based on total structure elucidations; however, it is still a challenge to acquire their X-ray quality single crystals and there are also many difficult questions still to be answered in this field, such as whether the face centered-cubic (fcc) structure in bulk gold would be preserved in these ultrasmall nanoclusters and how the organic ligands influence the configuration of the gold kernel, even for systems with the same nuclearity.^6^ Compared to the rapid progress of gold–thiolate nanoclusters starting from the seminal X-ray structure identification of the Au_102_ cluster,^7^ phosphine-protected gold nanoclusters are severely underdeveloped,^8^ although the first X-ray single crystal structure of a phosphine-protected cluster (*i.e.* Au_11_) dates back to 1969.^9^ The well-recognized phosphine-protected gold nanoclusters include undecagold Au_11_ and icosahedral Au_13_ protected by phosphine and halide.^10^

**Scheme 1:**
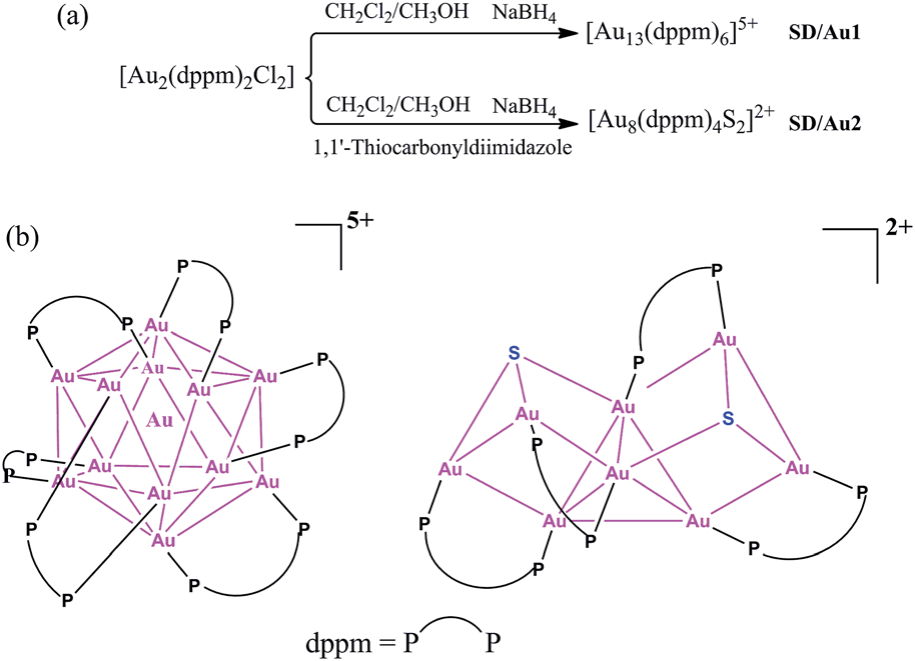
(a) Synthetic routes for [Au_13_(dppm)_6_]^5+^ and [Au_8_(dppm)_4_S_2_]^2+^ and (b) a chemical structure illustration of the trideca- and octagold nanoclusters.

Larger nanoclusters such as [Au_20_(PPhpy_2_)_10_Cl_4_] and [Au_20_(PP_3_)_4_] were recently characterized based on new phosphine ligands [PPhpy_2_ = bis(2-pyridyl)-phenylphosphine; and PP_3_ = tris[2-(diphenylphosphino)ethyl]phosphine].^11^ Among many of the reported gold nanoclusters, Au_13_ can be seen as a common ‘seed’ in the growth of larger ones from monoicosahedral Au_13_, biicosahedral Au_25_, to triicosahedral Au_37_, *i.e.* a ‘cluster of clusters’ motif.^12^ Of note, the configuration of the Au_13_ kernel usually deviates from that of the ideal *I*_h_ icosahedron depending on the protecting ligands; at the same time, such a variation in the metallic kernel may dramatically influence the physical and chemical properties.^13^ With these considerations in mind, herein, we revisited the dppm-protected gold nanoclusters and isolated two new members, [Au_13_(dppm)_6_]^5+^ (**SD/Au1**) and [Au_8_(dppm)_4_S_2_]^2+^ (**SD/Au2**), in this family (Scheme 1). Although **1** initially looks like its cousin with nitrate counteranions reported in 1981 (ref. 14) at first glance, in **1** the metallic kernel severely deviates from an ideal *I*_h_ icosahedron by the elongation of three Au–Au contacts to approximately 3.5 Å, giving it *C*_3_ symmetry. After introducing a S^2–^ releasing reagent, another novel octanuclear gold nanocluster was isolated that possessed a heart-shaped *C*_2_ symmetric Au_8_S_2_ core (central Au_4_ tetrahedron + two Au_2_S units).

## Results and discussion

### The X-ray structures of [Au_13_(dppm)_6_]Cl_5_ (**SD/Au1·5Cl**) and [Au_8_(dppm)_4_S_2_]Cl_2_ (**SD/Au2·2Cl**)

Details of the synthesis are shown in the ESI.† Briefly, to 12 mL of methanol and dichloromethane suspension containing 0.02 mmol Au_2_(dppm)_2_Cl_2_,^15^ a freshly prepared solution of NaBH_4_ (0.60 mmol in 1 mL of cold water) was added dropwise under vigorous stirring. The color immediately changed from colorless to dark green, and then to black. The reaction continued for 12 h at 0 °C in air under the exclusion of light. The crude black solid obtained by rotary evaporation was dissolved in 6 mL of CH_2_Cl_2_, and the resulting solution was subject to diffusion of benzene to afford black crystals (Scheme S1†). The identity was determined to be [Au_13_(dppm)_6_]Cl_5_ (**SD/Au1·5Cl**). Similarly, in the presence of a S^2–^ releasing reagent during a similar synthesis as for **SD/Au1·5Cl**, we could obtain [Au_8_(dppm)_4_S_2_]Cl_2_ (**SD/Au2·2Cl**) as yellow block crystals. The difference in the kernel structures between the two systems may arise from the etching effect of S^2–^ on the Au core surface due to its strong coordination ability toward Au atoms. Therefore, the introduction of S^2–^ in the synthesis of Au nanoclusters can significantly influence the final core structure.

**Fig. 1:**
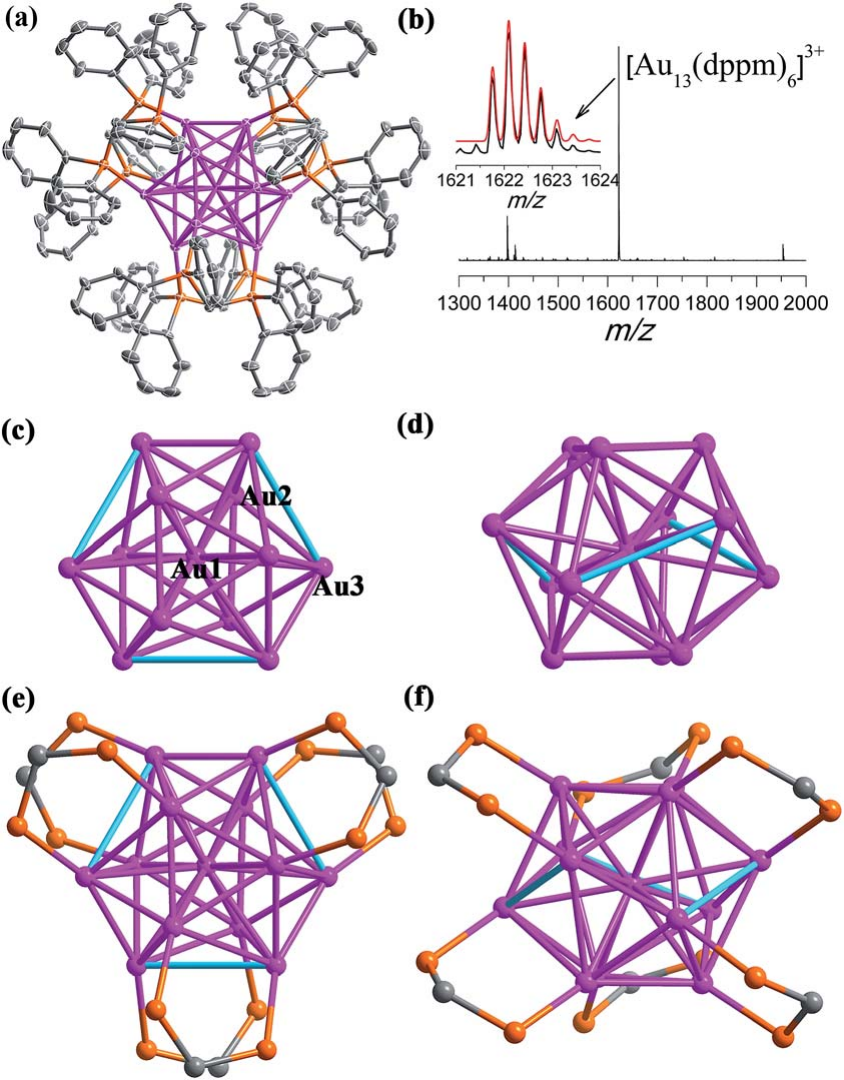
(a) The X-ray crystal structure of cationic [Au_13_(dppm)_6_]^5+^. Thermal contours are drawn at the 50% probability level. Color labels: purple, Au; orange, P; and gray, C. H atoms and Cl^–^ ions are omitted. (b) The positive mode of the HRESI-MS of **SD/Au1·5Cl** dissolved in CH_2_Cl_2_. Inset: comparison of the measured (black trace) and simulated (red trace) isotopic patterns of [Au_13_(dppm)_6_]^3+^. (c and e) Top view of the kernel structure of Au_13_ along the *C*_3_ axis with the crystallographically unique Au atoms labelled. (d and f) Side view of the kernel structure of Au_13_. The three cyan edges denote the longer Au–Au separations (3.4995(5) Å).

The single crystal X-ray diffraction (SCXD) analysis of **SD/Au1·5Cl** at 173 K indicates that it crystallizes in the trigonal space group *P*31*c* and that the asymmetric unit contains only one sixth of the Au_13_ nanocluster. The overall structure of **SD/Au1** is a distorted icosahedral Au_13_ kernel with six μ_2_-dppm ligands bound to its six edges of the outer shell (Fig. 1a). The Au1 (denoted as Au_c_ hereafter) site possesses one *C*_3_ axis and 3 *C*_2_ axes, giving it an occupancy of 1/6, whereas all of the Cl^–^ ions are located on the *C*_3_ axis and Cl2 additionally sits on the inversion center. Thus, in total, five uncoordinated Cl^–^ ions are found in the lattice corresponding to each Au_13_ cluster, making the charge state of the cationic part of **SD/Au1** +5. As shown in Fig. 1b, the positive mode of the high-resolution electrospray ionization mass spectrometry (HR-ESI-MS) shows a main peak centered at *m*/*z* 1622.0950 corresponding to [Au_13_(dppm)_6_]^3+^ (calcd. 1622.0949). The different charge state between the X-ray diffraction and mass spectroscopy results may be attributed to the trapping of two additional electrons by **SD/Au1** under the mass spectroscopy conditions.

The views of the Au_13_ kernel of **SD/Au1** drawn orthogonal with respect to the *C*_3_ axis are shown in Fig. 1c–f. The Au_13_ kernel consists of Au_3_ trigons perpendicular to the *C*_3_ axis at two poles and a chair-like Au_6_ ring (or Au_7_ if considering the central Au as well) sandwiched by them at the equatorial position. Each dppm ligand bridges two Au atoms, one at the pole and another at the equator (Au–P: 2.282(2) and 2.346(2) Å). The distortion of the Au_13_ kernel in **SD/Au1** is mainly reflected by three extraordinarily long Au–Au contacts of nearly 3.5 Å and a larger acute bond angle around the central Au atom (73.519(12)°), which makes the symmetry of the metallic kernel differ substantially from that of an ideal *I*_h_ icosahedron. The central Au atom (Au_c_) binds to twelve surface Au atoms (Au_s_), giving twelve radial bonds of 2.7024(4) and 2.9236(4) Å, whereas 27 of the 30 Au_s_–Au_s_ bonds fall in the range of 2.7155(7)–3.0272(5) Å, leaving the remaining three Au_s_–Au_s_ separations to be 3.4995(5) Å, which are much longer than those observed in many other phosphine-protected Au nanoclusters and thus do not belong in the classic bonding range for aurophilic interactions.^16^ Although phosphines are deemed as one of the best types of ligand to stabilize gold nanoclusters, and the one mostly used is triphenylphosphine,^17^ diphosphine-protected (hereafter Ph_2_P(CH_2_)_*m*_PPh_2_ = L^*m*^ for short) gold nanoclusters with atomically-precise structural identification are still few, such as [Au_11_(L^2^)_6_]^3+^,^18^ [Au_13_(L^2^)_5_Cl_2_]^3+^,19*a* [Au_6_(L^3^)_4_]^2+^,^20^ [Au_8_(L^3^)_4_]^2+^,^21^ [Au_11_(L^3^)_5_]^3+^,^22^ and the largest is [Au_22_(L^8^)_6_].^23^ Dppm, as the shortest diphosphine ligand, has rarely been used to build gold nanoclusters; only two were reported previously, Au_5_ and Au_13_.^14^

The most interesting feature of **SD/Au1·5Cl** compared to the previous [Au_13_(dppm)_6_](NO_3_)_4_ 14 structure is the configuration of the Au_13_ kernel, which is an opened icosahedral cage in **SD/Au1** instead of a closed one. For a perfect icosahedron, it should have twofold, threefold, and fivefold rotation axes, but the latter is missing in **SD/Au1** due to the distortion. It is also worth noting that in [Au_13_(dppm)_6_](NO_3_)_4_, both the Au_s_–Au_s_ and Au_c_–Au_s_ bond lengths are below 3.0 Å (2.75–2.98 Å). By comparing opened icosahedral **SD/Au1** and closed icosahedral [Au_13_(dppm)_6_](NO_3_)_4_ and [Au_13_(dppe)_5_(C≡CPh)_2_]^3+^,19*b* we found that **SD/Au1** and [Au_13_(dppm)_6_](NO_3_)_4_ have the same ligand shell but different electron counts in the Au_13_ core, whereas **SD/Au1** and [Au_13_(dppe)_5_(C≡CPh)_2_]^3+^ have different ligand shells but isoelectronic Au_13_ cores, which facilitated us to reasonably assign the distortion of the Au_13_ core to the synergistic effects of the electron count of the Au_13_ core and ligand size as well as the ligand arrangement on the surface. The current Au_13_ cluster carries five positive charges with eight valence electrons delocalized in “superatomic orbitals” (1S^2^1P^6^), which matches a major shell closing in the electron shell model.^24^ Thus, **SD/Au1·5Cl** is the first gold nanocluster with an opened icosahedral Au_13_ kernel.

Considering the high oxidation state of **SD/Au1**, we also tried to reduce it by adding NaBH_4_ in CH_2_Cl_2_ in the presence of ^*n*^Bu_4_NPF_6_, and as a result we isolated another Au_13_^5+^ cluster, **SD/Au3·4PF_6_·Cl**, which has almost the same structure as that of **SD/Au1·5Cl**, except for the difference in the counteranions. The synthesis and structural graphic of **SD/Au3·4PF_6_·Cl** are shown in the ESI.† These results indicate that an 8e Au_13_^5+^ nanocluster cannot be reduced to a 10e Au_13_^3+^ nanocluster and in turn support the oxidation state of **SD/Au1**. Due to the large thermal ellipsoids of two of the four PF_6_^–^ in the X-ray structure of **SD/Au3·4PF_6_·Cl**, we re-determined the number of PF_6_^–^ using ^31^P NMR of HCl-digested **SD/Au3·4PF_6_·Cl** (Fig. S2†), which clearly showed the ratio of dppm (singlet, *δ* = 28.40 ppm) and PF_6_^–^ (heptet, *δ* = –133.36, –137.01, –140.80, –144.19, –147.41, –151.17, and –154.62 ppm) to be 1 : 0.31 (calcd. 1 : 0.33), indicating a total of four PF_6_^–^ anions in **SD/Au3** and verifying the +5 oxidation state of **SD/Au3**.

**Fig. 2:**
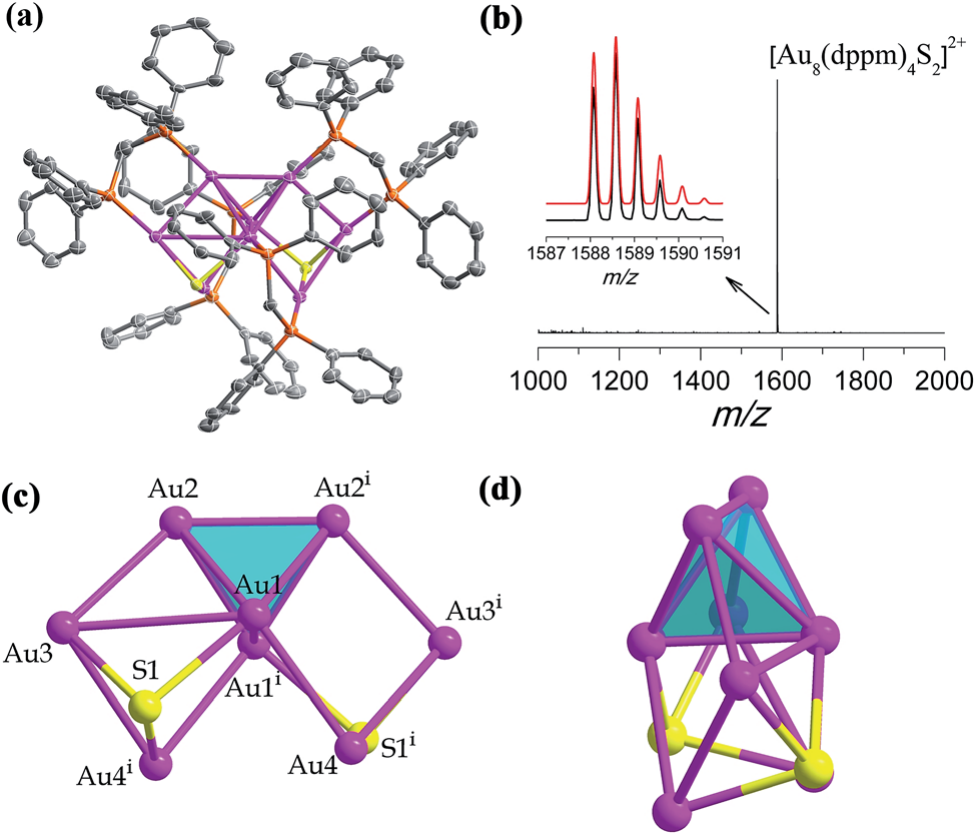
(a) The X-ray crystal structure of cationic [Au_8_(dppm)_4_S_2_]^2+^. Thermal contours are drawn at the 50% probability level. Color labels: purple, Au; orange, P; and gray, C. H atoms and Cl^–^ ions are omitted. (b) The positive mode of the HRESI-MS of **SD/Au2** dissolved in CH_2_Cl_2_. Inset: comparison of the measured (black trace) and simulated (red trace) isotopic patterns of [Au_8_(dppm)_4_S_2_]^2+^. (c and d) Orthogonal views of the kernel structure of Au_8_S_2_.

In the presence of a S^2–^ releasing reagent during a similar synthesis as for **SD/Au1·5Cl**, we isolated crystals of **SD/Au2·2Cl** in benzene or CH_2_Cl_2_ solvent. The single crystal structure analysis of **SD/Au2·2Cl** at 173 K reveals that it crystallized in an orthorhombic *Ccca* space group with a half molecule in the asymmetric unit. This nanocluster contains eight Au atoms, four dppm ligands, and two S^2–^ ions (Fig. 2a). The composition and charge state were further confirmed by the good comparison of the HR-ESI-MS which gave one intense peak at *m*/*z* 1588.5774 that is assigned to the parent [Au_8_(dppm)_4_S_2_]^2+^ with a simulated isotopic distribution pattern and no other fragments were observed in the *m*/*z* range of 1000–2000 (Fig. 2b). A *C*_2_ axis passes through the Au_8_ cluster through the mid points of Au1–Au1^*i*^ and Au2–Au2^*i*^ (symmetric code: *i* = –*x* + 1.5, –*y* + 1, *z*). **SD/Au2** is not a Au-centered polyhedral skeleton but has a “core + 4*exo*” structure exhibiting a heart shape. The octanuclear kernel in **SD/Au2** has a tetrahedral core and two pairs of *exo* gold atoms attached at the opposite edges through edge-sharing, thus being described as a Au_4_ + 2(Au_2_) type structure, as shown in Fig. 2c and d. The four *exo* Au atoms form rectangles by sharing an edge of the Au_4_ tetrahedron. The pure metallic kernel is further capped by two μ_3_-S^2–^ ions with Au–S distances of 2.297(4)–2.600(4) Å, giving the overall core structure of Au_8_S_2_. The Au–Au bonds within the tetrahedron are in the range of 2.6223(13)–2.8155(13) Å, which are shorter than that in metallic gold (2.88 Å). The core-to-*exo* distances (Au1–Au4 = 2.9907(9) Å and Au2–Au3 = 2.9269(9) Å) are markedly longer than those in the tetrahedron. Each dppm ligand as a bidentate bridge links one Au atom in the tetrahedron and one in an *exo* position to consolidate this “core + 4*exo*” structure (Au–P: 2.263(4)–2.320(4) Å). Several Au_8_ nanoclusters with different kernel geometries have been previously reported, such as capped centered chair-like [Au_8_(PPh_3_)_7_]^2+^ 25a and [Au_8_(PPh_3_)_8_]^2+^,25*b* [bitetrahedron + two]-type [Au_8_(dppp)_4_Cl_2_]^2+^,^21^ [Au_8_(dppp)_4_(C≡CPh)_2_]^2+^,^26^ and tritetrahedral [Au_8_(dppp)_4_]^2+^;^21^ however, a “core + 4*exo*” structure type has not been isolated hitherto. The closest case is [Au_8_(PMes_3_)_6_]^2+^ reported by Sharp *et al.* in 1994,^27^ and it also has a tetrahedral core like in **SD/Au2** but with two pairs of Au atoms appended on the vertices, and not the edges, of the tetrahedron. The current Au_8_ cluster carries two positive charges, thus the total number of free valence electrons is calculated to be 2 (*n* = 8 – 4 – 2), the smallest magic electron count. The formation of such a special Au_8_ cluster different from the other Au_8_ cousins found before may be caused by the coordination of the S^2–^ ligand, and its stability should be attributed to the jellium electronic shell closing of 1s^2^.

### The optical properties and time-dependent DFT (TDDFT) calculations of **SD/Au1·5Cl** and **SD/Au2·2Cl**

**Fig. 3:**
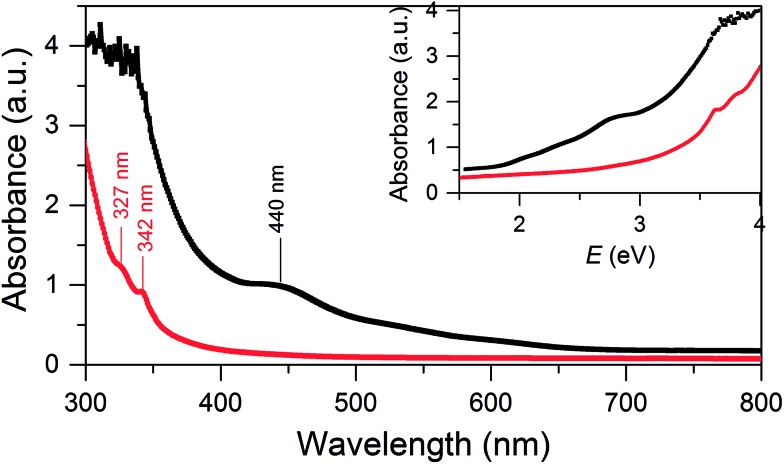
UV-vis absorption spectra of the Au nanoclusters **SD/Au1** (black) and **SD/Au2** (red). Inset: optical absorbance *vs.* photon energy (eV).

**SD/Au1·5Cl** can be dissolved in CH_2_Cl_2_ and ethanol, whereas **SD/Au2·2Cl** can be dissolved in methanol, acetonitrile and CH_2_Cl_2_. In these solvents, both **SD/Au1** and **SD/Au2** keep their cluster integrity without any disassembly, as shown by their parent ion peaks in the HR-ESI-MS (Fig. S3†). As revealed by time-dependent UV-vis spectra, no obvious changes were observed after their solutions were stored under ambient conditions for two weeks (Fig. S4†), indicating that both **SD/Au1** and **SD/Au2** are quite stable in solution. The optical absorption spectra of **SD/Au1** and **SD/Au2** were recorded in CH_2_Cl_2_ solution and are shown in Fig. 3. The absorption spectrum of **SD/Au1** showed tail-and-hump spectral features that comprised a strong absorption band in the UV region and a relatively weak shoulder peak at 440 nm tailing to the red region of the spectrum, whereas only two peaks at 327 and 342 nm were observed in the absorption spectrum of **SD/Au2** and no obvious peaks could be detected in the visible region. As discussed by Konishi,10*b* the absorption spectra of gold clusters are mainly influenced by the kernel nuclearity but far less so by the phosphine ligands. As shown in Table S7,† the absorption bands of Au_13_ clusters reported by Mingos and Konishi are very similar at ∼340 and ∼430 nm, whereas the absorption peak of **SD/Au1** shifted to 440 nm, which suggested that the absorption spectra of gold clusters are not only influenced by the kernel nuclearity but also the configuration of the core even with the same nuclearity.

**Fig. 4:**
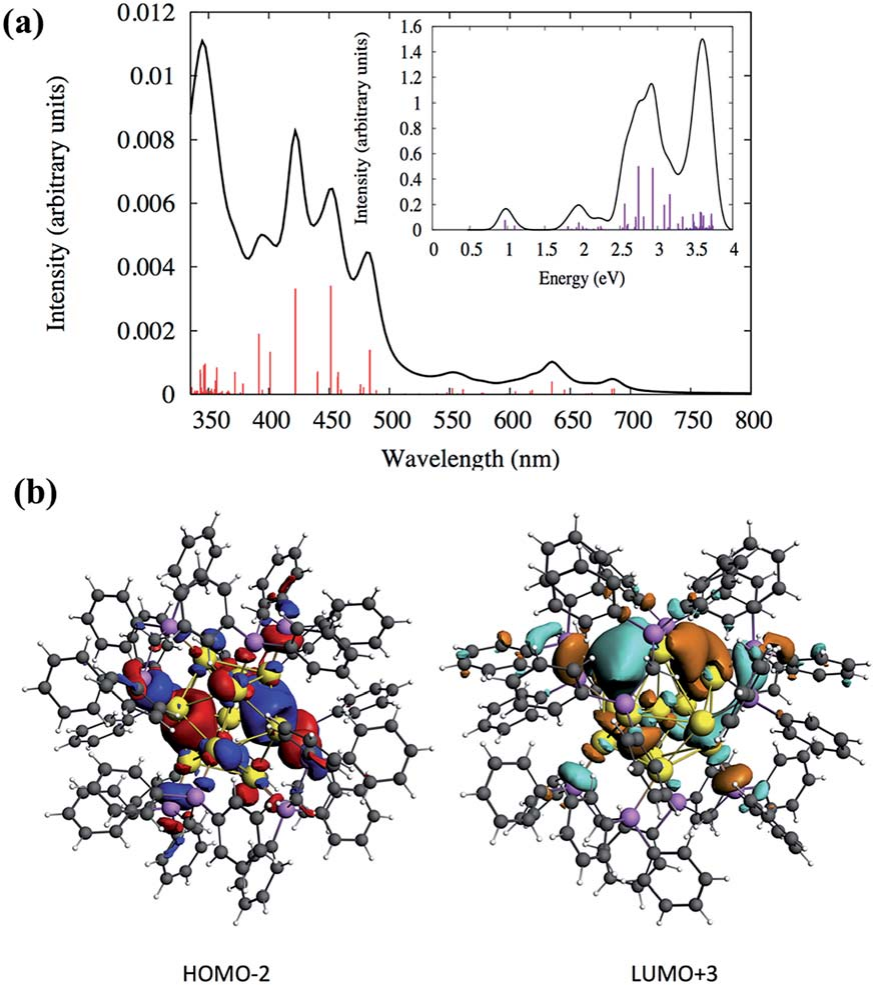
(a) Calculated TDDFT UV-vis absorption spectra of the Au nanocluster **SD/Au1**. Inset: optical absorbance *vs.* photon energy (eV). (b) The calculated HOMO–2 and LUMO+3 orbitals of **SD/Au1**.

Density functional theory (DFT) calculations were performed to analyze the electronic structures and optical absorptions of the clusters **SD/Au1** and **SD/Au2**. The calculated time-dependent DFT (TDDFT) spectrum for **SD/Au1** is shown in Fig. 4a. According to Fig. 4a and Table S3,† it is clear that there is a set of high and medium intensity peaks in the range of 401–484 nm (2.5–3 eV). The experimental peak observed at 440 nm is also clearly visible in the calculated absorption spectrum with an oscillator strength of 0.021–0.023 (Table S3†). The most probable transitions involved in the excitation around 440 nm are HOMO–2 → LUMO+2 and HOMO–2 → LUMO+3 with other transitions being less significant. The HOMO–2, LUMO+2, and LUMO+3 orbitals are gold core-based 6sp mixed orbitals with small contributions from the p orbitals from phosphorus (Fig. 4b). The HOMO–2 orbital has superatomic P character, as expected from the electron count of 8 in the core; similarly, the LUMO+3 has superatomic D-like character. A few low energy peaks were also observed in the calculated spectrum between 500–700 nm (1.7–2.4 eV) which could correspond to the very small peaks observed experimentally in a similar energy range. All of the relevant most probable transitions are shown in Table S3.† Apart from that, the theoretical calculation could identify a peak around ∼0.97 eV (1278 nm) which is the HOMO → LUMO transition (Fig. 4a and Table S3†). The HOMO and LUMO orbitals arise from gold 6s orbitals based in the core.

**Fig. 5:**
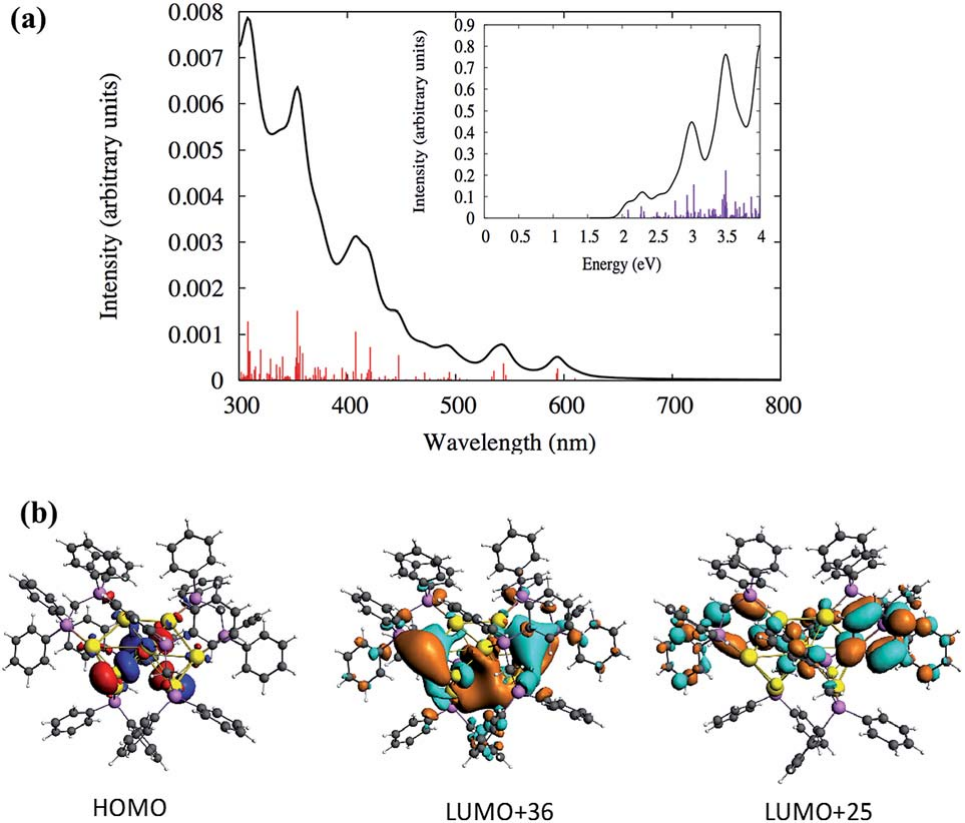
(a) Calculated TDDFT UV-vis absorption spectra of the Au nanocluster **SD/Au2**. Inset: optical absorbance *vs.* photon energy (eV). (b) The calculated HOMO, LUMO+36 and LUMO+25 orbitals of the cluster **SD/Au2**.

Similarly, the TDDFT absorption spectrum for the cluster **SD/Au2** was also analyzed, as shown in Fig. 5 and Table S4.† The experimental absorption spectrum could only identify two peaks around 327 and 342 nm, whereas the theoretically calculated spectrum demonstrates similar high intensity peaks around the same energy range (Table S4†). According to the calculated spectrum (Fig. 5 and Table S4†), a high intensity peak is predicted to arise around 408 nm (∼3.0 eV) with a high oscillator strength of 0.033 which has HOMO → LUMO+25 (Table S4†) as the most probable transition contributing to that peak. Due to the underestimation expected for the level of theory used in this work, this peak could correspond to the 342 nm peak observed experimentally. The deviation in the peak positions is similar to the underestimation/overestimation observed for this level of theory in previous work.^28^ This HOMO → LUMO+25 transition originates out of an orbital based on atomic p orbitals from sulfur atoms mixed with d orbitals of the gold core into the π* orbitals of the phenyl rings (Fig. 5b). The HOMO is not a superatomic orbital because the superatomic S orbital (corresponding with 2 core electrons) usually lies lower in energy than the gold d and ligand atomic orbitals.

From Fig. 5a, two other high intensity peaks are visible around 308 nm (4.0 eV) and 354 nm (3.5 eV) with higher oscillator strengths. The peak around 308 nm has HOMO–7 → LUMO+5 and HOMO → LUMO+36 as the most probable transitions. The occupied orbitals have p atomic orbital character from sulfur which is mixed with d orbitals of the gold core while the unoccupied orbitals have π* character from the phenyl rings which is mixed with the p and s orbitals of gold and p of phosphorus and sulfur. Fig. 5b shows the shapes of some orbitals involved in the transitions. The HOMO–1 → LUMO+34 and HOMO–2 → LUMO+24 transitions are mainly contributing to the peak at 345 nm. Here, the HOMO–2 and HOMO–1 have the p character from phosphorus and sulfur and have a small contribution from the d orbitals of the gold core atoms. The unoccupied orbitals have π* character from the phenyl rings with the p and s orbitals of gold mixed with p of phosphorus. The experimental spectrum did not identify any peaks in the visible range (400–700 nm) for the cluster **SD/Au2**. Even so, the calculated spectrum for **SD/Au2** shows that several other weak peaks are located at 421, 447, 544 and 594 nm, although these may be too broadened to be noticeable in experiment. All of the relevant transitions are shown in Table S4.†

### The photoluminescence properties of **SD/Au2·2Cl**

**Fig. 6:**
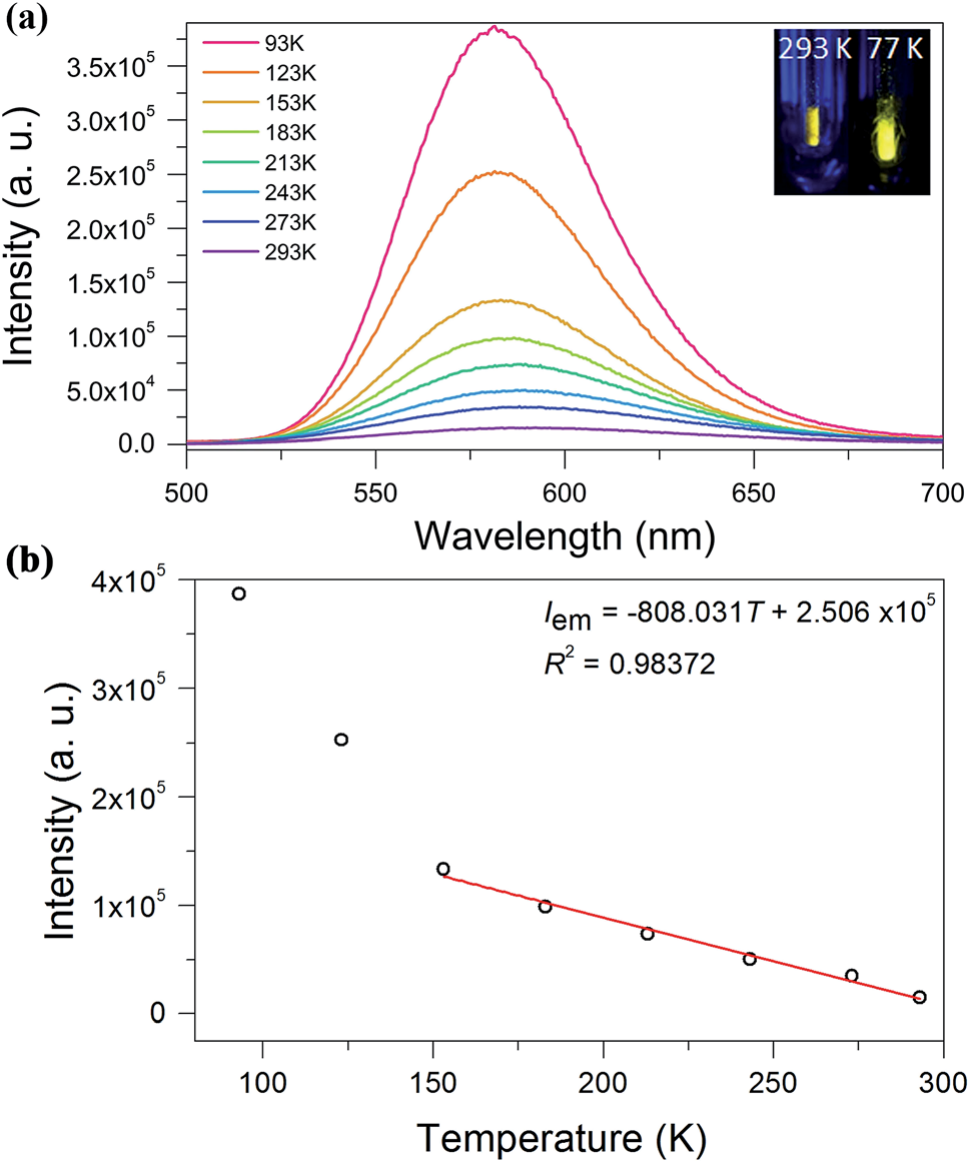
(a) The luminescence spectra of the cluster **SD/Au2** recorded in the solid state from 293 to 93 K under an excitation of 370 nm. Inset in the top right corner: photographs of the solid sample of **SD/Au2** excited by a hand-held UV lamp (365 nm) at room temperature and at 77 K. (b) The correlation between the temperature and emission intensity at 153–293 K.

We investigated the photoluminescence properties of **SD/Au1·5Cl** and **SD/Au2·2Cl** in the solid state at room temperature, and only **SD/Au2·2Cl** was actively luminescent, displaying yellow emission at 591 nm under an excitation of 370 nm (Fig. 6a). The absolute luminescence quantum yield obtained by using an integrating sphere is about 4.57% with the emission decay lifetimes on the microsecond scale (*τ*_1_^293 K^ = 1.43 and *τ*_2_^293 K^ = 7.10 μs), indicating a phosphorescent nature (Fig. S5†). Upon gradual cooling to 93 K, the emission maxima blue-shift from 591 to 581 nm along with a 3-fold intensity enhancement. The yellow emission can be tentatively assigned to a S^2–^ (or dppm) → Au charge transfer triplet state, probably with some mixing of a metal-centered (ds/dp) state.^29^ A linear correlation with the function of *I*_em_ = –808.031*T* + 250 600 and a correlation coefficient of 0.98 was constructed between the emission intensity and temperature (Fig. 6b). To clarify the mechanism of the temperature-dependent emission behaviors of **SD/Au2·2Cl**, we collected variable-temperature SCXD data for the same crystal at 93, 183, 243, 273, and 293 K (Table S5†). The results firstly ruled out the possible phase transition dependent emission changes as indicated by the similar unit cell parameters as well as the invariable space group. Upon cooling, we found that the average Au–Au bond distances in **SD/Au2** stay almost constant within the margin of error in the 93–293 K range (Fig. S6†). Such small variations of the Au–Au separation may not significantly influence the aurophilicity-related metal-centered (ds/dp) state, thus no red-shifted emission upon cooling was observed. Therefore, the cooling-induced emission blue-shift and intensity boosting should be caused by the enhanced rigidity of the Au_8_ cluster, which is in turn supported by the elongated lifetime (*τ*_1_^93 K^ = 10.08 and *τ*_2_^93 K^ = 19.60 μs) at low temperature.^30^ Compared to non-emissive Au_13_ with the same dppm protection shell, we can find that both the core structures and the incorporation of S^2–^ can affect the radiative path and efficiency of the luminescence.^31^

### Electrochemistry of **SD/Au1·5Cl** and **SD/Au2·2Cl**

**Fig. 7:**
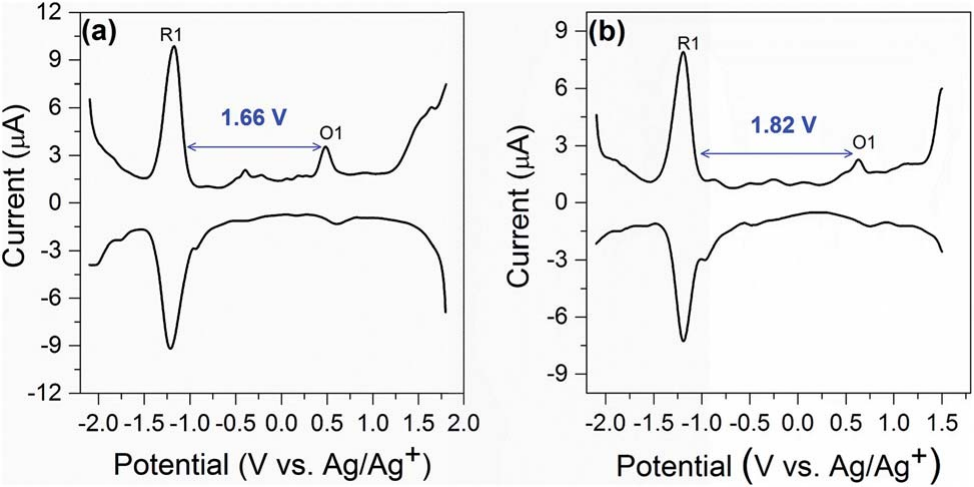
Differential pulse voltammetry spectrum of **SD/Au1·5Cl** (a) and **SD/Au2·2Cl** (b).

Electrochemical study can provide rich information related to the properties of highly monodisperse metal nanoparticles, especially with regards to HOMO–LUMO energy gaps determined from the difference value between the first oxidation and reduction peaks in differential pulse voltammograms or, equivalently, the *E*_1/2_ potentials for these redox couples in cyclic voltammograms.^32^ Differential pulse voltammetry (DPV) was performed to investigate the electrochemical properties of the nanocluster **SD/Au1·5Cl** and **SD/Au2·2Cl** at room temperature in CH_2_Cl_2_ solution (0.1 M ^*n*^Bu_4_NPF_6_ as electrolyte). As shown in Fig. 7a, the electrochemical energy gap of 1.66 V is in excellent agreement with that derived from the UV-vis spectroscopy data of 1.68 eV (440 nm) for **SD/Au1·5Cl**. We note that such an energy gap of Au_13_ is 0.10 eV smaller than that of a fully-closed icosahedral Au_13_(PPh_3_)_4_(SC_12_H_25_)_2_Cl_2_ cluster.^33^ The energy gap deduced from the DPV (Fig. 7b) of the Au_8_ cluster is 1.82 V, which is significantly smaller than 2.86 eV (342 nm) deduced from the experimental UV-vis spectrum of **SD/Au2·2Cl**. The theoretical calculation for cluster **2** predicted a HOMO–LUMO gap of 2.019 eV using the LB94/DZ level of theory. An optical gap of 2.032 eV is calculated for the first excited energy state (S_1_ state). Due to its low oscillator strength, it is not observed in the experimental spectrum, but lies close to the energy gap deduced from DPV. The difference between the electrochemical gaps and optical absorption of **SD/Au2·2Cl** is ascribed to the fact that the lowest energy excited state is not observed in the experimental UV-vis spectrum.^34^ The electrochemical energy gap of the Au_13_ cluster is smaller than that of the Au_8_ cluster, suggesting that their electronic structures are distinctively different and depend on the nuclearity and geometry of the metallic cores.

## Conclusions

In conclusion, we have successfully synthesized two new small gold nanoclusters: [Au_13_(dppm)_6_]^5+^ and [Au_8_(dppm)_4_S_2_]^2+^. The atomically-precise structures of these two nanoclusters were determined by single-crystal X-ray crystallography. The frameworks of **SD/Au1** and **SD/Au2** contain an opened *C*_3_-symmetric icosahedral Au_13_ kernel and a heart-shaped *C*_2_ symmetric Au_8_S_2_ core with a new “core + 4*exo*” structure type, respectively. The correlations between the electronic structures and optical absorption spectra are revealed by TDDFT calculations. The stability of the Au_13_ and Au_8_ nanoclusters can be ascribed to 8- and 2-electron superatoms with 1S^2^1P^6^ and 1S^2^ configurations, respectively. More interestingly, the cluster **SD/Au2** exhibits bright yellow luminescence and a temperature-induced hypsochromic shift. This study thus not only enriches the structures of ultrasmall gold nanoclusters but also provides fundamental insights into their electronic structures and luminescence properties.

## Conflicts of interest

There are no conflicts to declare.

## Supplementary Material

Supplementary information

Crystal structure data

Crystal structure data
