# Supplementary material for: Diphosphine-protected ultrasmall gold nanoclusters: opened icosahedral Au13 and heart-shaped Au8 clusters
Source: Chem Sci. 2017 Dec 4;9(5):1251–8. doi: 10.1039/c7sc03566g (PMC5885941; doi:10.1039/c7sc03566g)

Electronic Supplementary Material (ESI) for Chemical Science.
This journal is © The Royal Society of Chemistry 2017

Electronic Supplementary Information (ESI)

Diphosphine-Protected Ultrasmall Gold Nanoclusters: Opened Icosahedral Au_{13} and Heart-Shaped Au_8 Clusters

Shan-Shan Zhang,[a, ǂ] Lei Feng,[a, ǂ] Ravithree D. Senanayake,[b, ǂ] Christine M. Aikens,[b] Xing-Po Wang,[a] Quan-Qin Zhao,[a] Chen-Ho Tung,[a] and Di Sun*,[a,c]

[a]*Key Lab of Colloid and Interface Chemistry, Ministry of Education, School of Chemistry and Chemical Engineering, Shandong University, Jinan, 250100, P. R. China.*

[b]*Department of Chemistry, Kansas State University, Manhattan, Kansas 66506, USA.*

[ǂ]*These authors contributed equally to this work.*

Experimental details

The precursor [$Au_2(dppm)_2Cl_2$] was synthesized via a literature procedure.[1] All reagents employed were commercially available and used as received without further purification. IR spectra were recorded on a PerkinElmer Spectrum Two in the frequency range of 4000-400 cm^{-1}. The elemental analyses (C and H) were determined on a Vario EL III analyzer. The diffuse-reflectance spectra were recorded on a UV/Vis spectrophotometer (Evolution 220, ISA-220 accessory, Thermo Scientific) using a built-in 10 mm silicon photodiode with a 60 mm Spectralon sphere. The excitation spectrum was recorded on a Lumina Fluorescence Spectrometer (Thermo Fisher) at the emission wavelength of 590 nm. Temperature-dependent photoluminescence measurements were carried out in an Edinburgh spectrofluorimeter (F920S) coupled with an Optistat DN cryostat (Oxford Instruments), and the ITC temperature controller and a pressure gauge were used to realize the variable-temperature measurement in the range of 80-300 K. Spectra were collected at different temperatures after a 5min homoiothermy. Time-resolved photoluminescence lifetime measurements were performed on the same instrument by using a time-correlated single-photon counting technique and the solid state quantum yields were determined on an Edinburgh FLS920 fluorescence spectrophotometer equipped with an integrating sphere. The high-resolution electrospray mass spectrometry was performed on an Agilent 6510Q-TOF mass spectrometer. Differential pulse voltammetry (DPV) was conducted on an electrochemical work station model CHI-660 with a standard three-electrode system (glassy carbon working, Pt wire auxiliary, and Ag/Ag^+ reference); this study was performed on CH_2Cl_2 solutions containing 0.1 M $NBu^n{}_4PF_6$ as supporting electrolyte in N_2 atmosphere. Inductively coupled plasma atomic emission spectroscopy was recorded in a Leeman ICP-AES Prodigy instrument for the elemental analysis of Au and P by digesting crystals in a 1:1 mixture of HNO_3 and HCl. The Au and P contents were analysed three times for each clusters with an average of the three readings presented herein. Energy-dispersive X-ray spectrum was measured using an SU-8010 field emission scanning electron microscope (FESEM; Hitachi Ltd., Tokyo, Japan)

equipped with an Oxford-Horiba Inca XMax50 energy-dispersive X-ray (EDX; Oxford Instruments Analytical, High Wycombe, England). ^{31}P NMR spectra were recorded in a J. Young NMR tube on Bruker Avance 500 spectrometers. (In general, 5 mg of freshly synthesized crystals of **SD/Au3** were digested by 0.1 mL 12 M HCl aqueous solution and 0.4 mL DMSO. The digestion solution was used directly for ^{31}P NMR measurement)

Computational details

Time dependent DFT (TDDFT) calculations were performed on the crystal structure coordinates of clusters **SD/Au1** and **SD/Au2** with the LB94[2]/DZ level of theory using the Amsterdam density functional (ADF)[3] software. ZORA[4] in ADF was used to treat scalar relativistic effects for gold. Plots of intensity vs. wavelength (nm) are fit with a Lorentzian with a full-width at half-maximum (FWHM) of 20 nm, whereas the intensity vs. energy (eV) spectra are fit with a Gaussian with a FWHM of 0.2 eV. The TDDFT calculations on both the clusters were analyzed to get the most probable transitions and the orbitals responsible for each prominent peak in the calculated optical absorption spectra. The most probable transitions were identified based on the oscillator strength values, weights and the transition dipole moment values. The orbitals were viewed from the ADF-GUI.

X-ray crystallography

Single crystals of **SD/Au1·5Cl** and **SD/Au2·2Cl·2CH$_2$Cl$_2$** with appropriate dimensions were chosen under an optical microscope and quickly coated with high vacuum grease (Dow Corning Corporation) to prevent decomposition. Intensity data and cell parameters were recorded at 173 K on a Bruker Apex II single crystal diffractometer, employing a Mo K_α radiation (λ = 0.71073 Å) and a CCD area detector. The raw frame data were processed using SAINT and SADABS to yield the reflection data file.[5] The structure was solved using the charge-flipping algorithm, as implemented in the program *SUPERFLIP*[6] and refined by full-matrix least-squares techniques against F_o^2 using the SHELXL program[7] through the OLEX2 interface.[8] Hydrogen atoms at carbon were placed in calculated positions and refined isotropically by using a riding model. Appropriate restraints or constraints were applied to the geometry and the atomic displacement parameters of the atoms in the cluster. All structures were examined using the Addsym subroutine of PLATON[9] to ensure that no additional symmetry could be applied to the models. Pertinent crystallographic data collection and refinement parameters are collated in Table S1. Selected bond lengths and angles are collated in Table S2.

A crystal of **SD/Au2·2Cl·4CH$_2$Cl$_2$** was attached to a nylon loop and mounted on a Rigaku Oxford Diffraction XtaLAB Synergy diffractometer equipped with a HyPix-6000HE area detector and an Oxford Cryosystems CryostreamPlus open-fow N_2 cooling device for variable-temperature data collection at 93, 183, 243, 273, 293 K, respectively.[10] The crystallographic data of **SD/Au3·4PF$_6$·Cl·C$_6$H$_6$** was collected with the same X-ray single crystal diffractometer. A preliminary set of cell constants and an orientation matrix were calculated from reflections harvested from a sampling of reciprocal space. Full data collections were carried out using Mo Kα radiation (λ = 0.71073 Å, PhotonJet-S Mo 50W Microfocus) with exposure times ranging from 0.5 to 2 seconds, frame widths of 1 degrees, and a detector distance of approximately 3.2 cm. The intensity data were scaled and corrected for absorption, and final cell constants were calculated from the xyz centroids of strong reflections from the actual data

collections after integration. Space group was determined based on systematic absences and intensity statistics. The structure was solved using the charge-flipping algorithm, as implemented in the program *SUPERFLIP*[6] and refined by full-matrix least-squares techniques against F_o^2 using the SHELXL program[7] through the OLEX2 interface.[8] Hydrogen atoms at carbon were placed in calculated positions and refined isotropically by using a riding model. Appropriate restraints or constraints were applied to the geometry and the atomic displacement parameters of the atoms in the cluster. Pertinent crystallographic data collection and refinement parameters are collated in Table S5. Selected bond lengths and angles are collated in Table S6.

References

1. Lin, Ivan J. B.; Hwang, J. M.; Feng, D.-F.; Cheng, M. C.; Wang, Y. *Inorg. Chem.* 1994, **33**, 3467-3472.
2. Van Leeuwen, R.; Baerends, E. *Phys. Rev. A.* 1994, **49**, 2421.
3. Te Velde, G.; Bickelhaupt, F. M.; Baerends, E. J.; Fonseca Guerra, C.; Gisbergen, S. J. A. van; Snijders, J. G.; Ziegler, T. *J. Comp. Chem.* 2001, **22**, 931-967.
4. Lenthe, E. v.; Baerends, E. J.; Snijders, J. G. *J. Chem. Phys.* 1993, **99**, 4597-4610.
5. *APEX3*, *SAINT* and *SADABS*. Bruker AXS Inc., Madison, Wisconsin, USA, 2015.
6. Palatinus, L.; Chapuis, G. *J. Appl. Crystallogr*. 2007, **40**, 786.
7. Sheldrick, G. M. *Acta. Crystallogr. Sect. C* 2015, **71**, 3.
8. Dolomanov, O. V.; Bourhis, L. J.; Gildea, R. J.; Howard, J. A. K.; Puschmann, H. *J. Appl. Crystallogr*. 2009, **42**, 339.
9. Spek, A. L. *Acta. Crystallogr. Sect*. D. 2009, **65**, 148.
10. Rigaku Oxford Diffraction. *CrysAlisPro Software system, version 1.171.39.7f,* Rigaku Corporation: Oxford, UK, 2015.

Synthesis of [Au_{13} (dppm)$_6$]·5Cl (SD/Au1·5Cl)

To a 12 mL solution (CH_2Cl_2-CH_3OH; v:v = 3:1) of [Au_2(dppm)$_2Cl_2$] (0.02 mmol, 24.6 mg) was added tetraphenylphosphonium chloride (0.02 mmol, 7.2 mg). The solution was vigorously stirred (1000 rpm) for 15 min. Then 25 μL triethylamine was added in. After another 15 min stirring, 1 mL $NaBH_4$ aqueous solution (24 mg/mL cold water) was added slowly in the above mixture under vigorous stirring (1500 rpm). The mixture reacted very fast and the color was immediately changed from colorless to dark green then to black. The reaction continued for 12 h at 0 °C. The mixture in the organic phase was dried by rotary evaporation to get black powder, which was washed by diethyl ether three times, giving a yield of 70%. The well-matched IR spectra between Au_{13} crystals and powders proved their homogeneity (see Fig. S9). The black powder was then dissolved in dichloromethane and the resulting dark-red solution was filtered through a syringe filter with a pore size of 450 nm and was diffused by benzene to obtain black crystals with a yield of 20 %. Anal. Calcd for $C_{150}H_{132}Au_{13}P_{12}Cl_5$ calcd (found): Au, 50.76 (50.99); P, 7.37 (7.12); C, 35.72 (35.66); H, 2.64 (2.58) %. IR: 3049 (w), 1484 (m), 1438 (m), 1186 (m), 1118 (m), 998 (w), 739 (m), 718 (s), 689 (s), 540 (s) 504 (s) cm^{-1}.

Synthesis of [Au_8(dppm)$_4S_2$]·2Cl·2CH_2Cl_2 (SD/Au2·2Cl)

An identical procedure to that detailed in the synthesis of **SD/Au1·5Cl** was used except for the addition of 1,1'-thiocarbonyldiimidazole (0.10 mmol, 17.8 mg) in the starting reaction. The yellow cystals could be isolated with a yield of 40 %. Anal. Calcd for $C_{102}H_{92}Au_8P_8S_2Cl_6$, calcd (found): Au, 46.10 (45.92); P, 7.25 (7.38); C, 35.84 (35.77); H, 2.71 (2.78) %. IR: 3049 (w), 2921 (m), 2850 (w), 1480 (w), 1436 (m), 1099 (m), 780 (w), 721 (m), 522 (s), 506 (s) cm^{-1}.

Synthesis of [Au_{13} (dppm)$_6$]·4PF_6·Cl·C_6H_6 (SD/Au3·4PF_6·Cl)

Typically, 27.7 mg of black powder of **SD/Au1·5Cl** was dissolved in 12 mL solution (CH_2Cl_2-CH_3OH; v:v = 3:1), and 7.7 mg tetra-n-butylammonium hexafluorophosphate was added in this solution. After stirring 10 min, 1 mL $NaBH_4$

aqueous solution (10 mg/mL cold water) was added slowly in the above mixture under vigorous stirring (1500 rpm). The reaction continued for 5 h at 0 °C. The mixture in the organic phase was dried by rotary evaporation to get black powder. The black powder was then dissolved in dichloromethane and diffused by benzene to obtain black crystals with a yield of 20 %. IR: 1441 (w), 1097 (w), 841 (s), 776 (m), 739 (m), 682 (m), 557 (m) 513 (s) cm^{-1}.

Table S1: Crystal data collection and structure refinement for SD/Au1·5Cl, SD/Au2·2Cl·2CH$_2$Cl$_2$ and SD/Au3·4PF$_6$·Cl·C$_6$H$_6$

Compound	**SD/Au1·5Cl**	**SD/Au2·2Cl·2CH$_2$Cl$_2$**	**SD/Au3·4PF$_6$·Cl·C$_6$H$_6$**
Empirical formula	$C_{150}H_{132}Au_{13}Cl_5P_{12}$	$C_{102}H_{92}Au_8Cl_6P_8S_2$	$C_{156}H_{138}Au_{13}ClF_{24}P_{16}$
Formula weight	5044.00	3418.07	5560.19
Temperature/K	173(2)	173(2)	93.00(10)
Crystal system	trigonal	orthorhombic	triclinic
Space group	*P*-31c	*C*cca	*P*-1
a/Å	19.878(2)	27.105(6)	17.4438(3)
b/Å	19.878(2)	29.820(7)	19.0667(4)
c/Å	27.361(3)	28.748(6)	24.5099(4)
α/°	90	90.00	86.3850(10)
β/°	90	90.00	84.3990(10)
γ/°	120	90.00	84.4130(10)
Volume/Å^3	9362(2)	23236(9)	8062.6(3)
Z	2	8	2
ρ_{calc}g/cm^3	1.789	1.954	2.290
μ/mm^{-1}	10.352	10.385	12.027
F(000)	4648.0	12720.0	5148.0
Radiation	MoKα (λ = 0.71073)	MoKα (λ = 0.71073)	MoKα (λ = 0.71073)
2Θ range for data collection/°	3.802 to 55.064	3.940 to 50	6.662 to 52.744
Reflections collected	7211	10236	148622
Independent reflections	7211	10236	32833
Data/restraints/parameters	7211/0/272	10236/12/568	32833/125/1820
Goodness-of-fit on F^2	0.981	1.038	1.023
Final R indexes [I>=2σ (I)]	R_1 = 0.0406, wR_2 = 0.0802	R_1 = 0.0542, wR_2 = 0.1277	R_1 = 0.0460, wR_2 = 0.0988
Final R indexes [all data]	R_1 = 0.0840, wR_2 = 0.0926	R_1 = 0.1037, wR_2 = 0.1547	R_1 = 0.0694, wR_2= 0.1087
Largest diff. peak/hole / e Å^{-3}	2.26/-1.50	3.48/-1.43	2.96/-2.40

Table S2: Selected bond lengths (Å) and angles (°) for SD/Au1·5Cl, SD/Au2·2Cl·2CH$_2$Cl$_2$ and SD/Au3·4PF$_6$·Cl·C$_6$H$_6$.

Compound **SD/Au1·5Cl**			
Au1—Au2^{i}	2.7024 (4)	Au1—Au3^{ii}	2.9236 (4)
Au1—Au2^{ii}	2.7024 (4)	Au2—Au2^{iv}	2.8457 (6)
Au1—Au2^{iii}	2.7024 (4)	Au2—Au2^{v}	2.8457 (6)
Au1—Au2^{iv}	2.7024 (4)	Au2—Au3^{ii}	3.0271 (6)
Au1—Au2	2.7023 (4)	Au2—Au3^{iv}	2.8326 (5)
Au1—Au2^{v}	2.7024 (4)	Au2—Au3	3.0021 (5)
Au1—Au3^{iv}	2.9236 (4)	Au2—P1	2.282 (2)
Au1—Au3	2.9236 (4)	Au3—Au2^{v}	2.8326 (5)
Au1—Au3^{iii}	2.9236 (4)	Au3—Au2^{ii}	3.0272 (5)
Au1—Au3^{i}	2.9236 (4)	Au3—Au3^{iii}	2.7155 (7)
Au1—Au3^{v}	2.9236 (4)	Au3—P2	2.346 (2)
Symmetry codes: (i) $-x+y, y, -z+3/2$; (ii) $x, x-y+1, -z+3/2$; (iii) $-y+1, -x+1, -z+3/2$; (iv) $-y+1, x-y+1, z$; (v) $-x+y, -x+1, z$.			
Compound **SD/Au2·2Cl·2CH$_2$Cl$_2$**			
Au2—Au2^{i}	2.6223 (13)	Au4—S1^{i}	2.306 (4)
Au2—Au3	2.9269 (9)	Au4—P9	2.265 (4)
Au2—P6	2.320 (4)	Au4—Au1	2.9907 (9)
Au2—S1	2.600 (4)	Au1—Au2^{i}	2.7827 (10)
Au2—Au1	2.6807 (9)	Au1—Au1^{i}	2.8155 (13)
Au3—Au4	3.0916 (10)	P1—Au1	2.289 (4)
Au3—S1^{i}	2.297 (4)	Au3—P8	2.263 (4)
P9—Au4—S1^{i}	172.44 (14)	P8—Au3—S1^{i}	173.95 (15)
P6—Au2—S1	98.80 (14)		
Symmetry code: (i) $-x+1/2, -y+1, z$.			
Compound **SD/Au3·4PF$_6$·Cl·C$_6$H$_6$**			
Au1—Au2	2.9159 (5)	Au4—P7	2.287 (3)
Au1—Au3	2.7027 (5)	Au5—Au6	3.0082 (5)
Au1—Au4	2.6967 (5)	Au5—Au11	2.8607 (6)
Au1—Au5	2.8855 (5)	Au5—Au13	2.6823 (6)
Au1—Au6	2.6991 (5)	Au5—P8	2.321 (3)
Au1—Au7	2.7065 (5)	Au6—Au7	2.8314 (6)
Au1—Au8	2.6955 (5)	Au6—Au8	2.8432 (5)
Au1—Au9	2.9762 (6)	Au6—Au12	3.0076 (6)
Au1—Au10	2.8744 (6)	Au6—Au13	2.8622 (5)
Au1—Au11	2.7089 (5)	Au6—P11	2.284 (3)
Au1—Au12	2.9726 (6)	Au7—Au8	2.8730 (6)
Au1—Au13	2.9195 (5)	Au7—Au10	3.0217 (5)

Au2—Au3	3.0367 (5)	Au7—Au13	3.0035 (5)
Au2—Au7	2.8528 (5)	Au7—P10	2.297 (3)
Au2—Au8	2.9458 (6)	Au8—Au9	2.9897 (6)
Au2—Au10	2.6885 (5)	Au8—Au12	2.8315 (6)
Au2—P2	2.333 (3)	Au8—P1	2.289 (3)
Au3—Au4	2.8595 (6)	Au9—Au12	2.6935 (6)
Au3—Au9	2.9903 (6)	Au9—P3	2.338 (3)
Au3—Au10	2.8583 (6)	Au10—Au11	3.0189 (6)
Au3—Au11	2.8490 (6)	Au10—P5	2.326 (3)
Au3—P4	2.289 (3)	Au11—Au13	3.0998 (6)
Au4—Au5	2.9714 (6)	Au11—P6	2.291 (3)
Au4—Au9	2.8220 (5)	Au12—P12	2.337 (3)
Au4—Au11	2.8525 (6)	Au13—P9	2.331 (3)
Au4—Au12	2.9805 (5)		

Table S3: The excited states, energies (in eV and nm), oscillator strengths, weights, transitions with the strongest weights, and the transition dipole moments involved in cluster SD/Au1 obtained by TDDFT calculations.

Excitation transitions	Energy (eV)	nm	Oscillator strength (a.u.)	Weight	Most weighted transitions	Transition dipole moment (a.u.)		
						X	Y	Z
1	0.969	1280	0.016	0.9896	HOMO → LUMO	2.2629	-0.2385	-0.0021
2	0.972	1276	0.017	0.9896	HOMO-1 → LUMO	-0.2371	-2.2632	0.0007
11	1.953	635	0.013	0.924	HOMO-10 → LUMO	1.1645	-0.0165	-0.0063
				0.0456	HOMO-11 → LUMO	-0.0039	-0.2492	-0.0001
12	1.953	635	0.011	0.8967	HOMO-11 → LUMO	0.0171	1.1053	0.0004
				0.0463	HOMO-10 → LUMO	0.2605	-0.0037	-0.0014
49	2.562	484	0.044	0.3557	HOMO-1 → LUMO+1	1.2486	0.45	0.2833
				0.316	HOMO → LUMO+2	1.1713	0.4229	-0.292
				0.1183	HOMO-45 → LUMO	-0.2116	-0.0009	0.0143
				0.0614	HOMO-1 → LUMO+2	0.1859	-0.5181	0.0175
				0.0484	HOMO → LUMO+1	0.1645	-0.4601	-0.0183
50	2.563	484	0.044	0.3729	HOMO-1 → LUMO+2	-0.4581	1.277	-0.0432
				0.2984	HOMO → LUMO+1	-0.4085	1.1424	0.0456
				0.0579	HOMO-1 → LUMO+1	0.5036	0.1815	0.1143
55	2.604	476	0.010	0.2979	HOMO-1 → LUMO+4	0.02	-0.3647	0.2288
				0.2143	HOMO → LUMO+3	0.0143	-0.2779	-0.1937
62	2.710	458	0.022	0.3472	HOMO-54 → LUMO	-0.0201	0.0004	0.2338
				0.2205	HOMO-52 →	0.1392	0.0002	0.0261

					LUMO			
				0.2017	HOMO- 2 → LUMO+2	0.3052	-0.0739	-0.0012
				0.0833	HOMO-1 → LUMO+3	0.1726	0.0078	-0.8194
				0.0773	HOMO → LUMO+4	-0.1712	-0.0111	-0.7882
63	2.713	457	0.017	0.3409	HOMO-54 → LUMO	-0.0199	0.0004	0.2315
				0.2265	HOMO-2 → LUMO+2	-0.3233	0.0783	0.0012
				0.2185	HOMO-52 → LUMO	-0.1385	-0.0002	-0.026
				0.0649	HOMO → LUMO+4	-0.1568	-0.0101	-0.7218
				0.0576	HOMO-1 → LUMO+3	0.1435	0.0065	-0.6811
68	2.815	440	0.023	0.6497	HOMO-2 → LUMO+3	0.0735	-1.9129	-0.0004
				0.2469	HOMO-1 → LUMO+5	0.0907	0.8718	0.0018
				0.0331	HOMO → LUMO+5	-0.321	0.0337	-0.0072
				0.0276	HOMO-2 → LUMO+4	0.3938	0.0154	-0.0042
69	2.816	440	0.021	0.6451	HOMO-2 → LUMO+4	1.9037	0.0746	-0.0203
				0.256	HOMO → LUMO+5	-0.8922	0.0936	-0.0201
75	3.091	401	0.042	0.8447	HOMO-2 → LUMO+5	0.0274	-0.0012	1.9098
				0.0382	HOMO → LUMO+7	0.0893	0.0142	-0.1901
				0.0375	HOMO-1 → LUMO+6	-0.0873	-0.0141	-0.1895
				0.0152	HOMO → LUMO+4	0.0711	0.0046	0.3274
				0.0146	HOMO-1 → LUMO+3	-0.0677	-0.0031	0.3215
79	3.165	392	0.060	0.4938	HOMO-1 → LUMO+6	0.3132	0.0505	0.6795
				0.3842	HOMO → LUMO+7	-0.2797	-0.0444	0.5955
				0.0487	HOMO-2 →	0.0065	-0.0003	0.4531

					LUMO+5			
80	3.275	379	0.011	0.9555	HOMO-60 → LUMO	0.0002	-0.0001	0.682
				0.0111	HOMO-2 → LUMO+5	0.0031	-0.0001	0.213
85	3.335	372	0.022	0.9245	HOMO-2 → LUMO+6	0.0764	1.3883	0.0005
86	3.335	372	0.022	0.9185	HOMO-2 → LUMO+7	1.3779	-0.0771	-0.0033
104	3.474	357	0.027	0.4197	HOMO-4 → LUMO+1	-0.2397	-0.0572	-0.8306
				0.2937	HOMO-3 → LUMO+2	0.1566	0.0591	-0.6837
109	3.485	356	0.014	0.803	HOMO-1 - LUMO+13	0.009	0.0086	0.5518
				0.0722	HOMO-4 → LUMO+1	0.0992	0.0237	0.3438
				0.0279	HOMO-5 → LUMO+1	0.1601	-0.0397	-0.0014
				0.0191	HOMO-3 → LUMO+2	-0.0399	-0.0151	0.1742
138	3.572	347	0.016	0.5633	HOMO-5 → LUMO+4	0.0018	-0.0703	0.0004
				0.1153	HOMO-4 → LUMO+3	0.3071	-0.012	-0.4871
				0.0934	HOMO-3 → LUMO+4	-0.2782	0.0108	-0.4464
				0.0382	HOMO-1 → LUMO+18	0.0507	0.0024	-0.1127
139	3.572	347	0.030	0.2947	HOMO-5 → LUMO+4	-0.0013	0.0508	-0.0003
				0.217	HOMO-4 → LUMO+3	0.4213	-0.0165	-0.6681
				0.1815	HOMO-3 → LUMO+4	-0.3879	0.015	-0.6222
				0.064	HOMO-1 → LUMO+18	0.0656	0.0031	-0.1458
				0.0273	HOMO-10 → LUMO+2	-0.0612	-0.0143	0.1114
140	3.583	346	0.029	0.3656	HOMO-9 → LUMO+1	0.1896	0.8376	0.0025
				0.3126	HOMO-9 → LUMO+2	0.7316	-0.1862	0.0119
				0.0382	HOMO →	0.0814	0.002	0.0221

					LUMO+19			
				0.0233	HOMO-3 → LUMO+4	0.1388	-0.0054	0.2226
				0.0203	HOMO-4 → LUMO+3	0.1287	-0.005	-0.204
141	3.583	346	0.029	0.369	HOMO-9 → LUMO+2	0.7949	-0.2022	0.013
				0.3104	HOMO-9 → LUMO+1	-0.1747	-0.7716	-0.0023
				0.0271	HOMO-4 → LUMO+4	-0.0059	-0.1507	0.0098
155	3.611	343	0.021	0.5541	HOMO-7 → LUMO+3	-0.1096	-0.0032	0.326
				0.1403	HOMO-10 → LUMO+2	-0.138	-0.0323	0.2514
				0.1156	HOMO-76 → LUMO	0.2381	0.0198	0.0127
				0.032	HOMO-11 → LUMO+1	0.0625	0.0109	0.1214
157	3.613	343	0.024	0.2738	HOMO-10 → LUMO+2	0.1927	0.0451	-0.351
				0.2308	HOMO-11 → LUMO+1	-0.1678	-0.0294	-0.3261
				0.1216	HOMO-7 → LUMO+3	-0.0514	-0.0015	0.1527
				0.0862	HOMO-76 → LUMO	0.2054	0.0171	0.011

Table S4: The excited states, energies (in eV and nm), oscillator strengths, weights, transitions with the strongest weights, and the transition dipole moments involved in cluster SD/Au2 obtained by TDDFT calculations.

Excited state	Energy (eV)	nm	Oscillator strength (a.u.)	Weight	Most weighted transitions	Transition dipole moments (a.u.)		
						X	Y	Z
**Sym B**								
3	2.278	544	0.012	0.9713	HOMO → LUMO+3	-0.8761	-0.3665	0
				0.0265	HOMO-1 → LUMO+2	0.1431	-0.0278	0
17	2.771	447	0.017	0.9857	HOMO-2 → LUMO+2	0.6527	0.9299	0
26	2.944	421	0.023	0.7436	HOMO-2 → LUMO+4	-0.7342	1.351	0
				0.1413	HOMO-1 → LUMO+21	0.0089	-0.1371	0
32	3.041	408	0.033	0.7105	HOMO → LUMO+25	-0.6624	-1.0242	0
				0.0682	HOMO-2 → LUMO+8	-0.024	-0.0487	0
				0.0537	HOMO → LUMO+26	0.1688	0.0001	0
58	3.482	356	0.024	0.4621	HOMO-2 → LUMO+24	0.1879	1.0714	0
				0.3379	HOMO-1 → LUMO+34	-0.5725	-0.4256	0
60	3.504	354	0.047	0.2498	HOMO-1 → LUMO+34	-0.4906	-0.3647	0
				0.2246	HOMO-2 → LUMO+24	-0.1306	-0.7445	0
61	3.515	353	0.016	0.875	HOMO-3 → LUMO+14	-0.0007	-0.2004	0
				0.0448	HOMO-2 → LUMO+24	-0.0582	-0.3319	0
				0.0308	HOMO-4 → LUMO+5	0.0674	-0.0335	0
				0.0186	HOMO-1 →	-	-0.0995	0

					LUMO+34	0.1338		
71	3.644	340	0.016	0.5551	HOMO-2 → LUMO+27	- 0.5927	-0.4899	0
				0.1507	HOMO → LUMO+35	0.1894	0.0922	0
76	3.706	335	0.011	0.4945	HOMO → LUMO+35	- 0.3403	-0.1656	0
				0.1404	HOMO-4 → LUMO+12	- 0.0795	-0.0125	0
				0.1166	HOMO-2 → LUMO+27	- 0.2694	-0.2227	0
				0.0753	HOMO-8 → LUMO+1	- 0.0483	0.0454	0
				0.0577	HOMO-3 → LUMO+23	- 0.0058	0.053	0
				0.0289	HOMO → LUMO+36	0.3307	0.1053	0
89	3.875	320	0.021	0.9165	HOMO-6 → LUMO+4	0.1457	-0.9607	0
				0.0353	HOMO-2 → LUMO+34	- 0.1788	0.1285	0
100	3.997	310	0.020	0.5732	HOMO-2 → LUMO+34	- 0.7095	0.51	0
				0.1389	HOMO-6 → LUMO+8	0.0461	0.0091	0
				0.0601	HOMO → LUMO+36	0.4596	0.1464	0
101	4.003	310	0.011	0.7683	HOMO-4 → LUMO+23	0.0575	-0.0509	0
				0.0556	HOMO → LUMO+36	- 0.4417	-0.1407	0
103	4.010	309	0.020	0.5261	HOMO-7 → LUMO+5	0.1858	0.0141	0
				0.1344	HOMO-4 → LUMO+23	-0.024	0.0213	0
				0.0908	HOMO → LUMO+36	- 0.5641	-0.1797	0
				0.0596	HOMO-2 → LUMO+34	- 0.2285	0.1642	0
106	4.022	308	0.040	0.3746	HOMO-7 → LUMO+5	- 0.1565	-0.0119	0
				0.1317	HOMO → LUMO+36	- 0.6783	-0.2161	0
				0.1236	HOMO-2 →	-	0.2361	0

					LUMO+34	0.3285		
**Sym A**								
2	2.087	594	0.008	0.9892	HOMO-1 → LUMO	0	0	-1.0086
58	3.457	359	0.019	0.558	HOMO → LUMO+34	0	0	-1.013
				0.1199	HOMO-4 → LUMO+4	0	0	0.2106
60	3.493	355	0.012	0.8145	HOMO-4 → LUMO+4	0	0	0.546
				0.0591	HOMO → LUMO+34	0	0	0.3281
81	3.768	329	0.015	0.4388	HOMO-1 → LUMO+35	0	0	-0.5028
				0.218	HOMO-5 → LUMO+11	0	0	0.0721

Table S5: Crystal data collection and structure refinement for SD/Au2·2Cl·2CH_2Cl_2 at five different temperatures.

Temperature/K	93	183	243	273	293
Crystal system	Orthorhombic				
Space group	*C*cce				
a/Å	29.8513(7)	29.9954(10)	30.1665(11)	30.1847(7)	30.1004(7)
b/Å	27.0724(7)	27.1880(9)	27.3016(10)	27.3736(7)	27.2979(7)
c/Å	28.9960(6)	29.1016(7)	29.2120(8)	29.2444(6)	29.2079(6)
Volume/$Å^3$	23433.0(10)	23732.8(13)	24058.9(14)	24163.7(10)	23999.5(10)
Z	8	8	8	8	8
ρ_{calc} g/cm^3	2.034	2.008	1.887	1.879	1.892
μ/mm^{-1}	10.391	10.260	10.030	9.987	10.055
F(000)	13392.0	13392.0	12720.0	12720.0	12720.0
Radiation	MoKα (λ = 0.71073)				
Reflections collected	57061	58728	59359	59897	58803
Independent reflections	11919	12062	12193	12245	12210
R_{int}	0.0699	0.0493	0.0535	0.0590	0.0699
R_{sigma}	0.0492	0.0394	0.0433	0.0476	0.0554
Data/parameters	11919/595	12062/580	12193/568	12245/568	12210/568
Goodness-of-fit on F^2	1.014	1.024	0.975	1.019	1.017
Final *R* indexes [I>=2σ (I)]	R_1 = 0.0418, wR_2 = 0.0968	R_1 = 0.0393, wR_2 = 0.0879	R_1 = 0.0396, wR_2 = 0.0837	R_1 = 0.0427, wR_2=0.0850	R_1 = 0.0536, wR_2 = 0.1024
Final R indexes [all data]	R_1 = 0.0552, wR_2 = 0.1033	R_1 = 0.0554, wR_2 = 0.0942	R_1 = 0.0601, wR_2 = 0.0911	R_1 = 0.0668, wR_2=0.0928	R_1 = 0.0870, wR_2 = 0.1154

Table S6: Selected bond lengths (Å) and angles (°) for SD/Au2·2Cl·$2CH_2Cl_2$ at five different temperatures.

**93 K**			
Au1—Au1^{i}	2.6227 (6)	Au2—P4	2.286 (2)
Au1—Au2^{i}	2.7927 (4)	Au3—Au4	3.1055 (4)
Au1—Au2	2.6811 (4)	Au3—S1^{i}	2.303 (2)
Au1—Au3	2.9371 (4)	Au3—P2	2.265 (2)
Au1—S1	2.5956 (19)	Au4—S1^{i}	2.300 (2)
Au1—P1	2.326 (2)	Au4—P3	2.262 (2)
Au2—Au2^{i}	2.8149 (6)	Au2—Au4	2.9876 (4)
P3—Au4—S1^{i}	172.38 (7)	P2—Au3—S1^{i}	175.17 (7)
P1—Au1—S1	97.53 (7)		
Symmetry code: (i) −*x*+1/2, −*y*, *z*.			
**183 K**			
Au1—Au1^{i}	2.6257 (5)	Au2—P4	2.2863 (18)
Au1—Au2^{i}	2.7927 (4)	Au3—Au4	3.1056 (4)
Au1—Au2	2.6811 (4)	Au3—S1^{i}	2.3026 (18)
Au1—Au3	2.9371 (4)	Au3—P2	2.2650 (19)
Au1—S1	2.5930 (17)	Au4—S1^{i}	2.2998 (18)
Au1—P1	2.3265 (18)	Au4—P3	2.2619 (19)
Au2—Au2^{i}	2.8149 (5)	Au2—Au4	2.9876 (4)
P3—Au4—S1^{i}	172.37 (6)	P2—Au3—S1^{i}	175.18 (6)
P1—Au1—S1	97.54 (6)		
Symmetry code: (i) −*x*+1/2, −*y*, *z*.			
**243 K**			
Au1—Au1^{i}	2.6321 (6)	Au2—P4	2.2878 (19)
Au1—Au2^{i}	2.7978 (4)	Au3—Au4	3.1149 (4)
Au1—Au2	2.6827 (4)	Au3—S1^{i}	2.302 (2)
Au1—Au3	2.9436 (4)	Au3—P2	2.267 (2)
Au1—S1	2.5982 (19)	Au4—S1^{i}	2.3032 (2)
Au1—P1	2.3297 (19)	Au4—P3	2.265 (2)
Au2—Au2^{i}	2.8244 (6)	Au2—Au4	2.9958 (4)
P3—Au4—S1^{i}	172.34 (7)	P2—Au3—S1^{i}	175.20 (7)
P1—Au1—S1	97.55 (6)		
Symmetry code: (i) −*x*+1/2, −*y*, *z*.			
**273 K**			
Au1—Au1^{i}	2.6312 (6)	Au2—P4	2.286 (2)
Au1—Au2^{i}	2.7949 (4)	Au3—Au4	3.1196 (4)
Au1—Au2	2.6825 (4)	Au3—S1^{i}	2.299 (2)
Au1—Au3	2.9461 (4)	Au3—P2	2.266 (2)

Au1—S1	2.596 (2)	Au4—S1^{i}	2.301 (2)
Au1—P1	2.329 (2)	Au4—P3	2.263 (2)
Au2—Au2^{i}	2.8266 (6)	Au2—Au4	2.9995 (4)
P3—Au4—S1^{i}	172.27 (7)	P2—Au3—S1^{i}	175.19 (7)
P1—Au1—S1	97.51 (7)		
Symmetry code: (i) −*x*+1/2, −*y*, *z*.			
**293 K**			
Au1—Au1^{i}	2.6236 (8)	Au2—P4	2.283 (3)
Au1—Au2^{i}	2.7894 (6)	Au3—Au4	3.1134 (6)
Au1—Au2	2.6752 (5)	Au3—S1^{i}	2.291 (3)
Au1—Au3	2.9396 (5)	Au3—P2	2.258 (3)
Au1—S1	2.586 (3)	Au4—S1^{i}	2.291 (3)
Au1—P1	2.323 (3)	Au4—P3	2.258 (3)
Au2—Au2^{i}	2.8214 (8)	Au2—Au4	2.9927 (6)
P3—Au4—S1^{i}	172.32 (10)	P2—Au3—S1^{i}	175.31 (10)
P1—Au1—S1			
Symmetry code: (i) −*x*+1/2, −*y*, *z*.			

Figure S1: The excitation spectrum of SD/Au2·2Cl in the solid state.

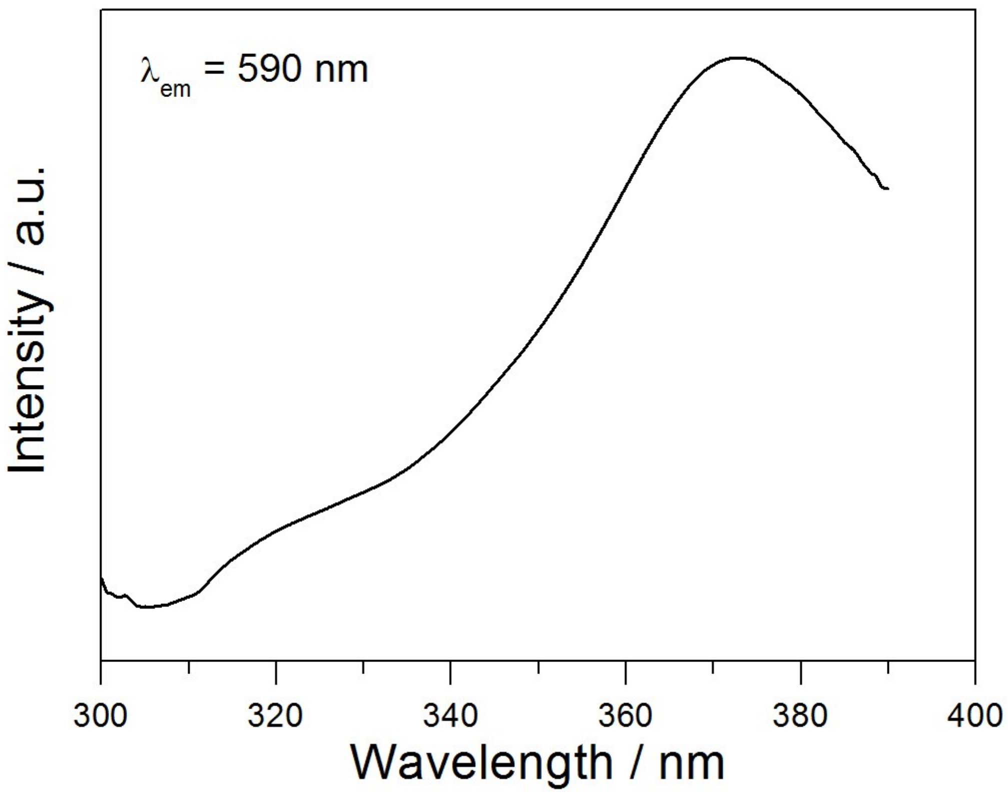

Figure S2: The ^{31}P NMR spectrum of SD/Au3·4PF_6·Cl in DMSO after HCl-digestion.

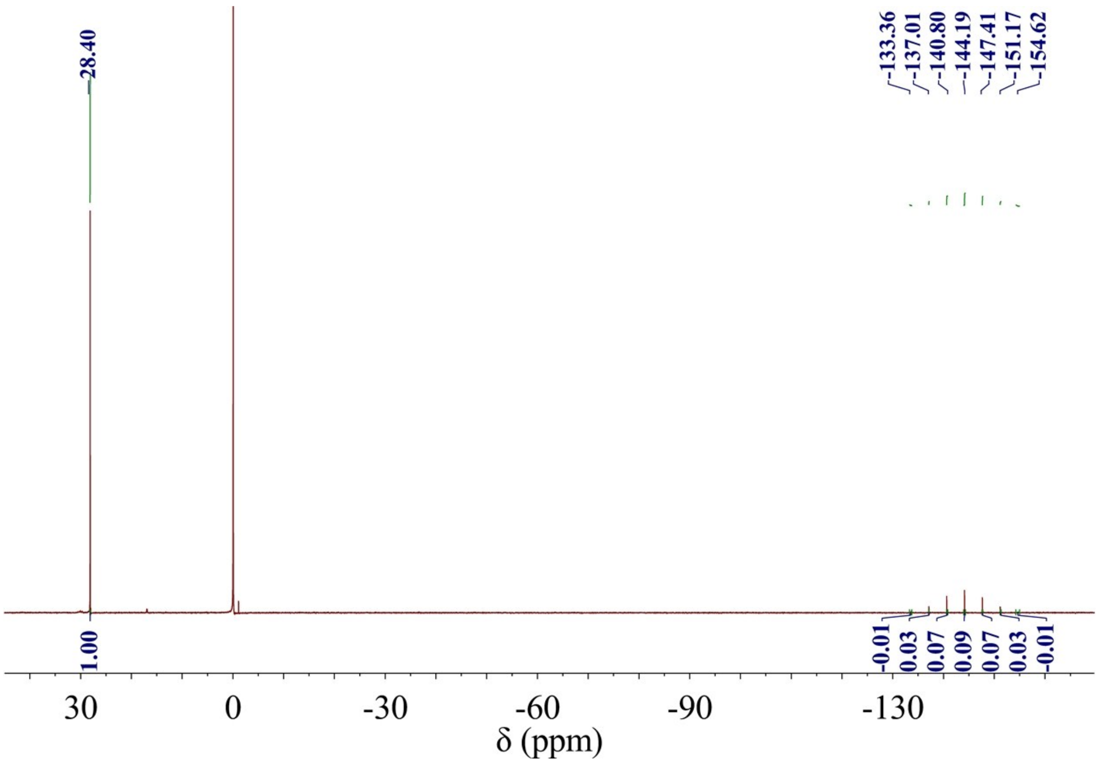

Figure S3: Positive ion mass spectra of SD/Au1·5Cl (a) and SD/Au2·2Cl (b) dissolved in different solvents.

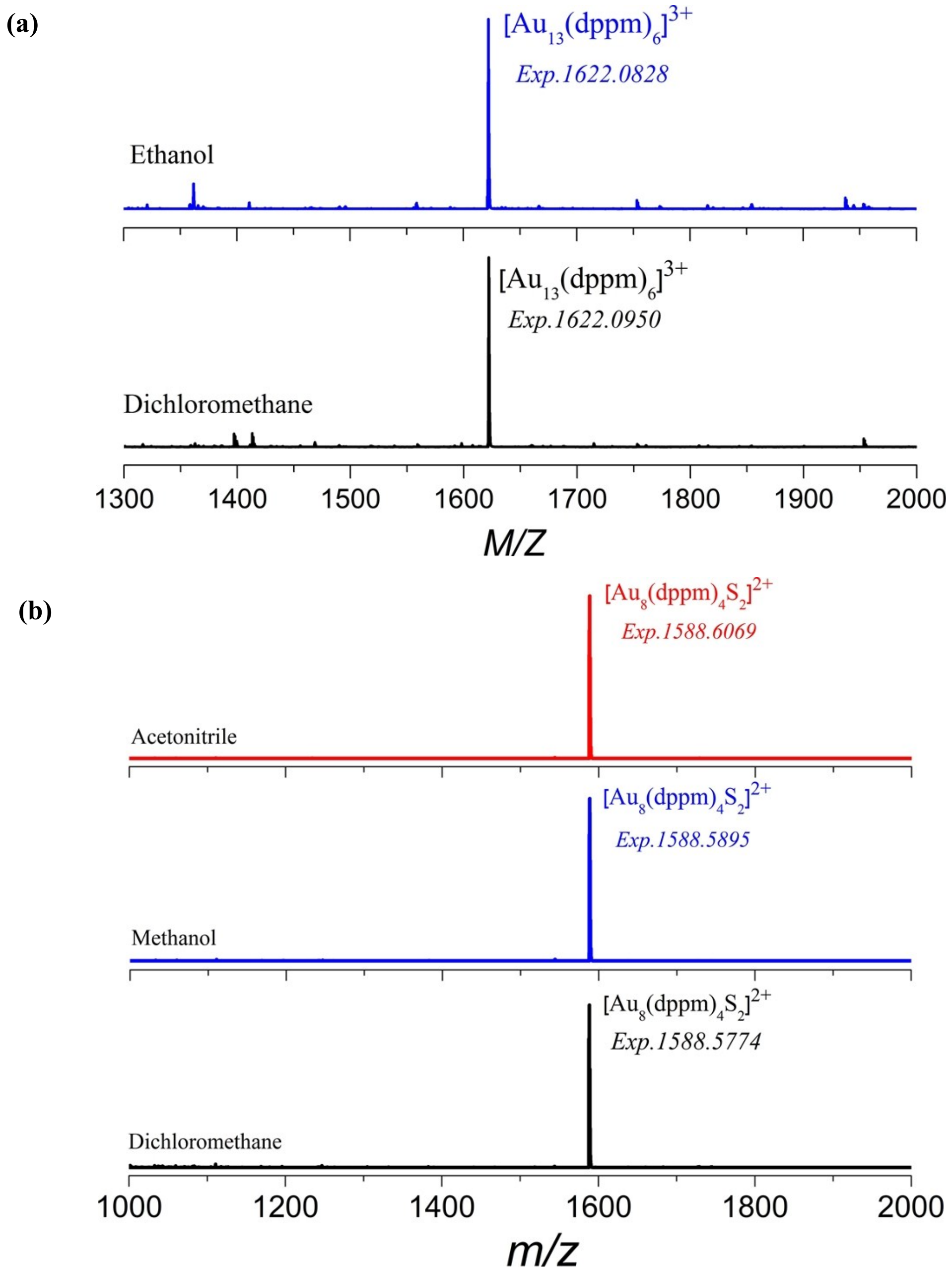

Figure S4: Time-dependent UV-vis absorption spectra of SD/Au1·5Cl (a) and SD/Au2·2Cl (b) in CH_2Cl_2.

(a)

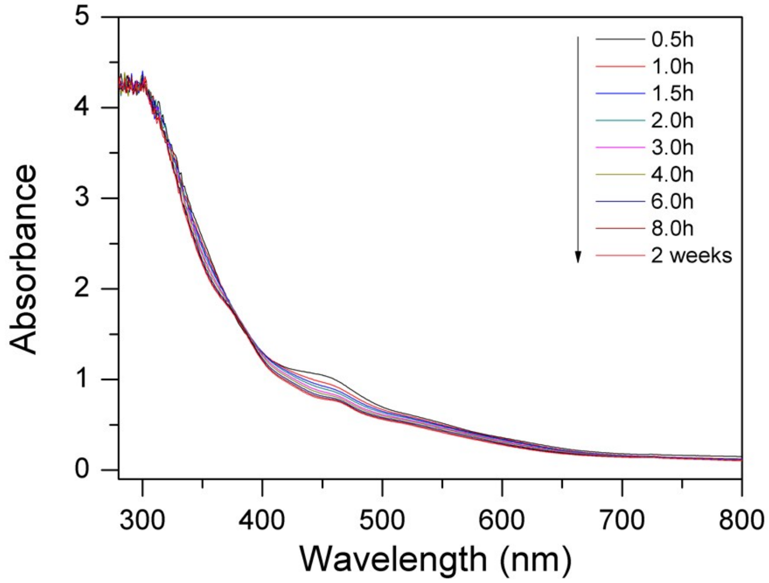

(b)

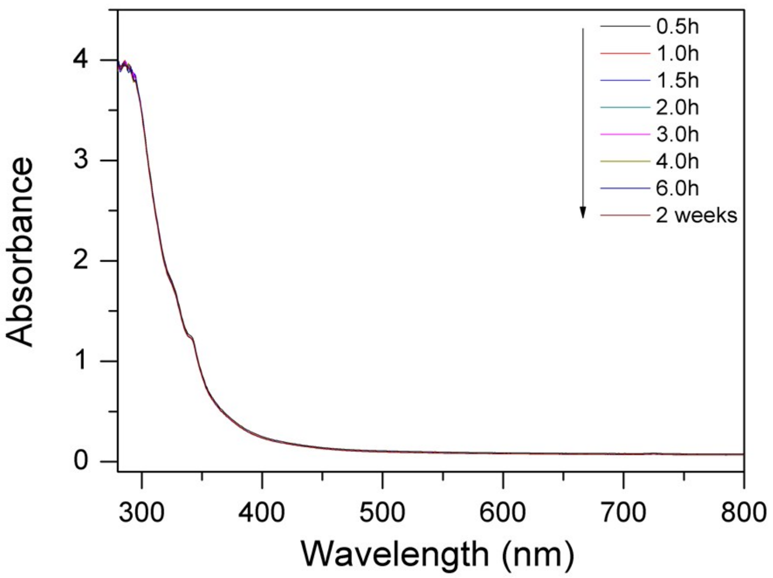

Figure S5: Luminescent lifetimes of SD/Au2·2Cl at 293 K (a) and 93 K (b) (red lines are fitting curves).

(a)

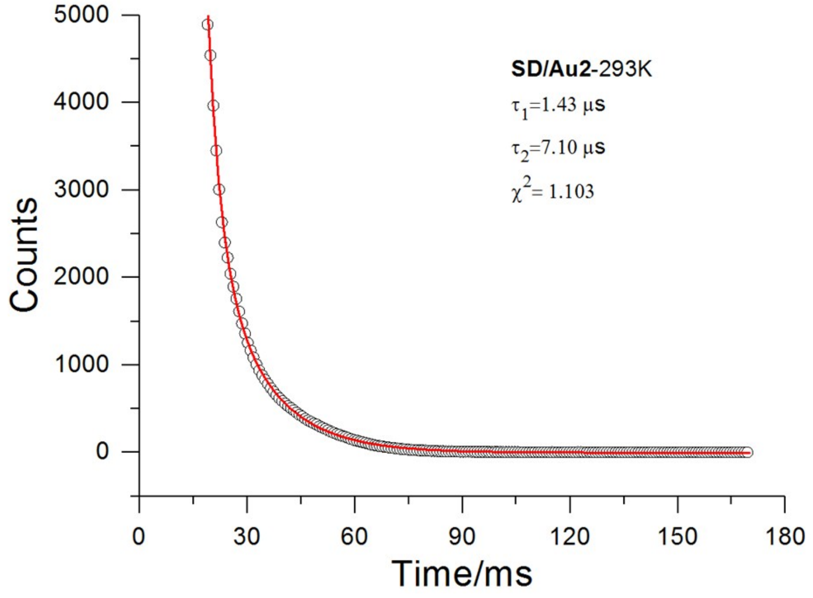

(b)

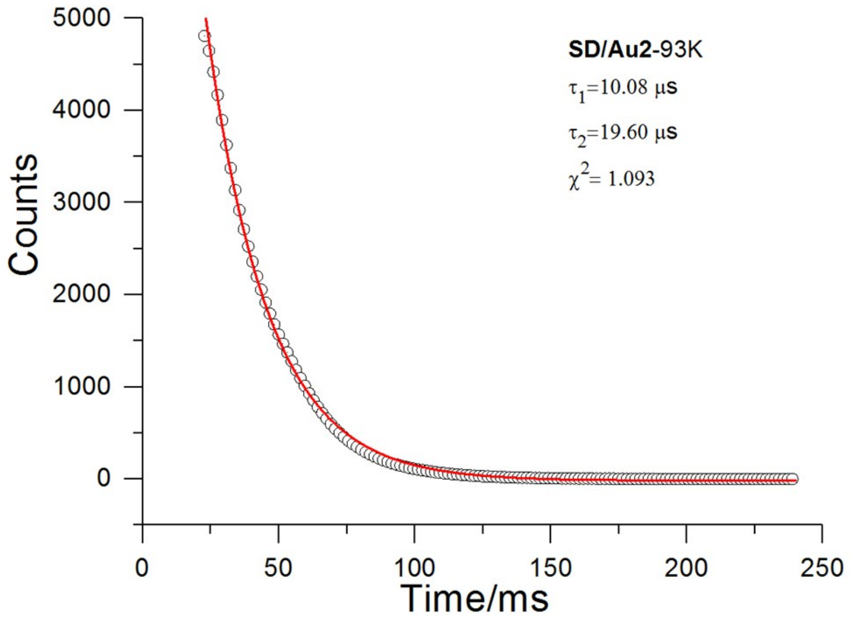

Figure S6: The evolution of average Au−Au bond distances in 93-293 K in SD/Au2.

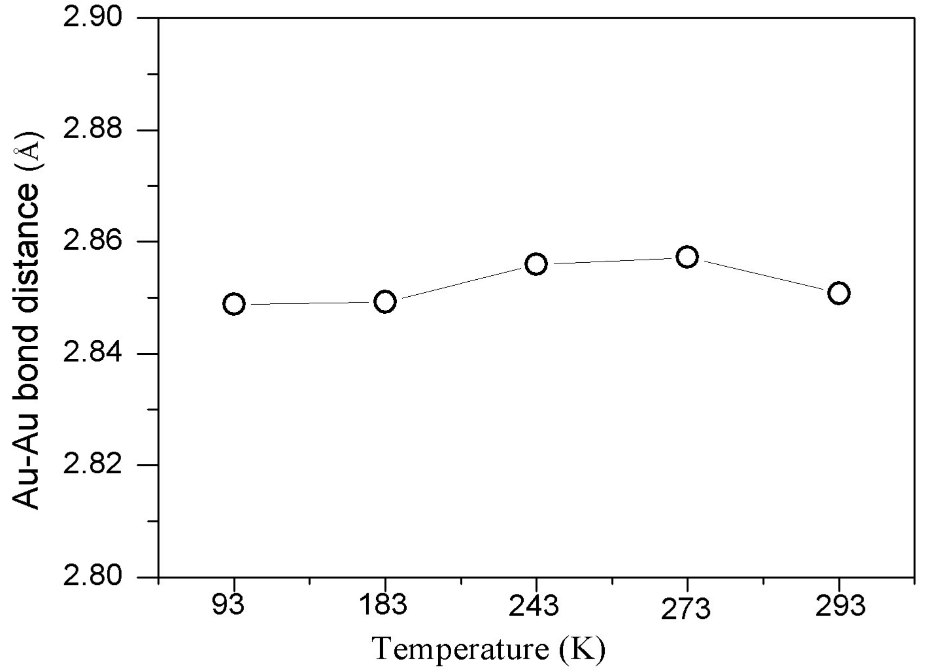

Figure S7: The energy dispersive spectroscopy (EDS) mapping of SD/Au1·5Cl.

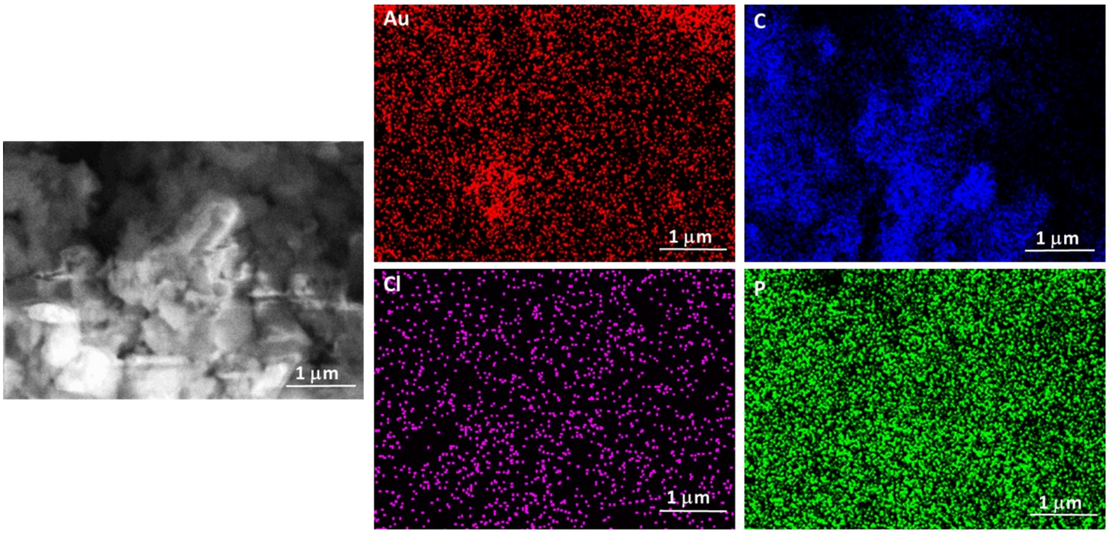

Figure S8: The energy dispersive spectroscopy (EDS) mapping of SD/Au2·2Cl.

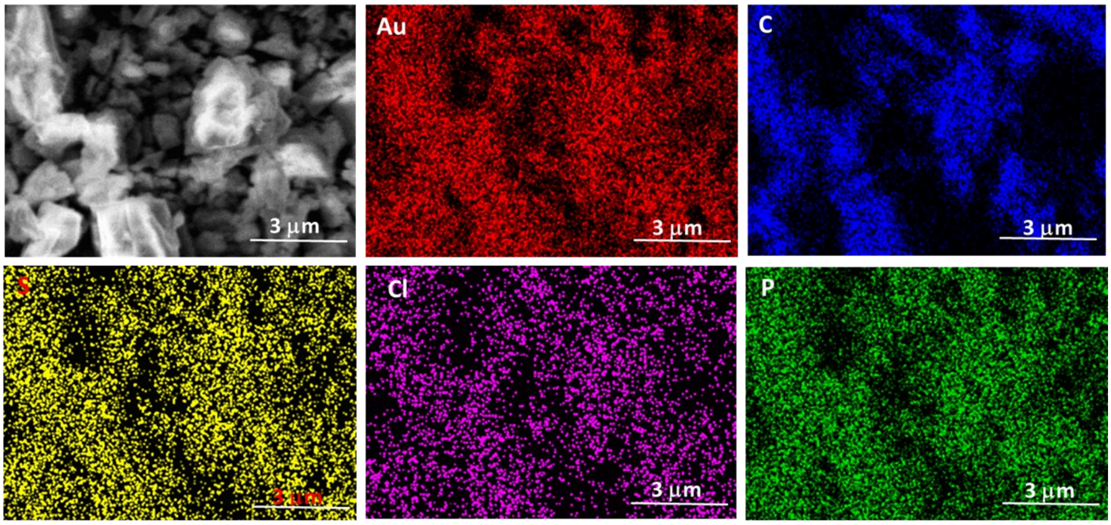

Figure S9: Compared IR spectra of crystals and bulk powders of SD/Au1·5Cl.

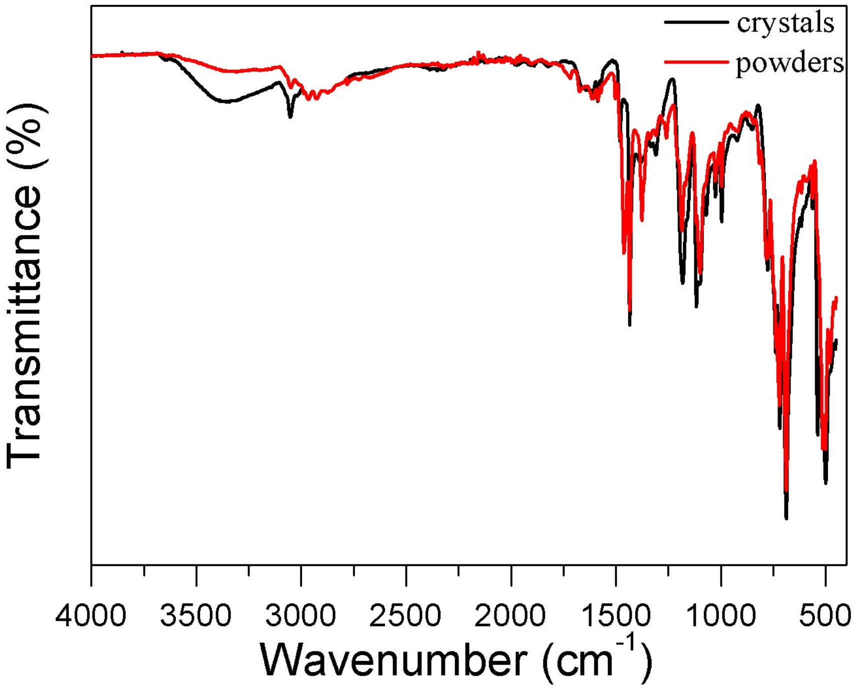

Figure S10: Photographs for crystals of SD/Au1·5Cl (a), SD/Au2·2Cl·2CH_2Cl_2 (b) and the bulk powder sample of SD/Au1·5Cl (c) taken by optical camera.

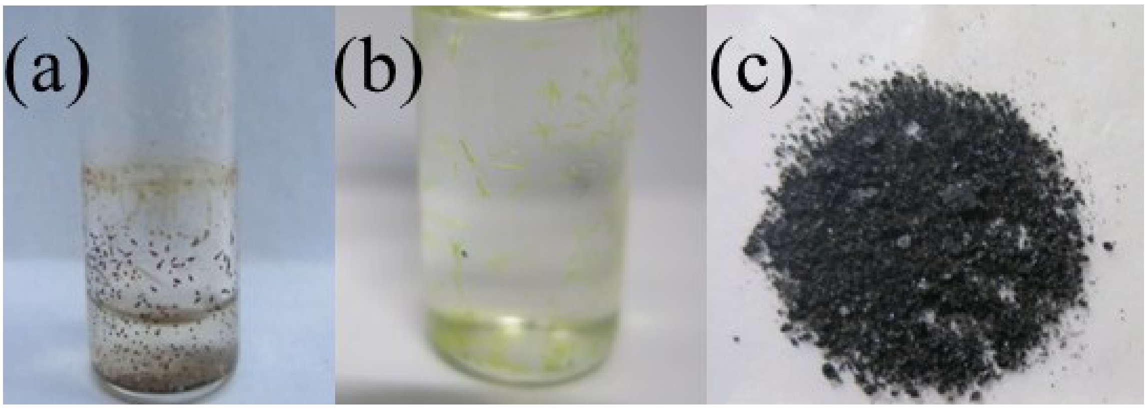

Figure S11: Two different views (a and b) of the structure of SD/Au3·$4PF_6$·Cl·C_6H_6. (H atoms are omitted; Occupancy: Cl1 0.65, Cl1A 0.35)

(a)

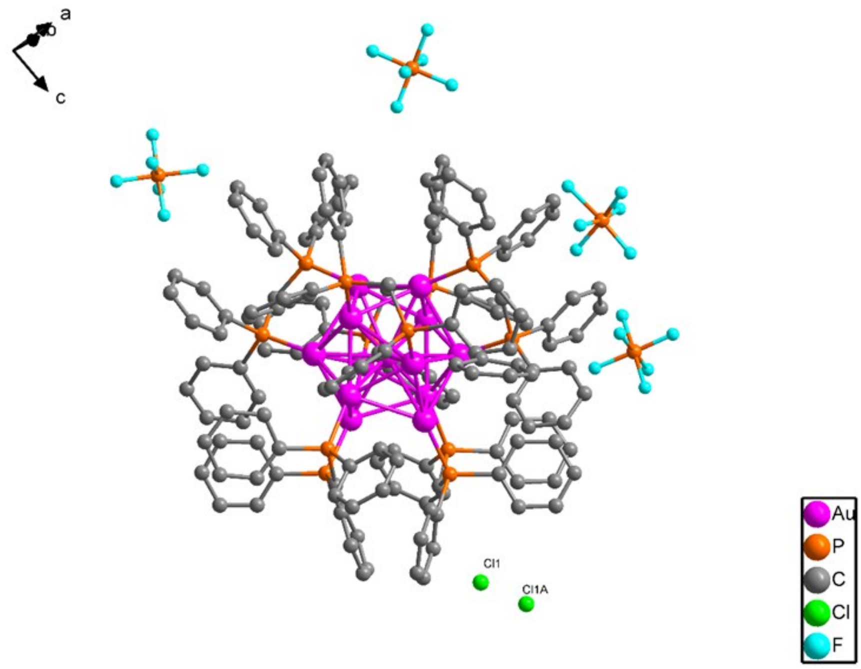

(b)

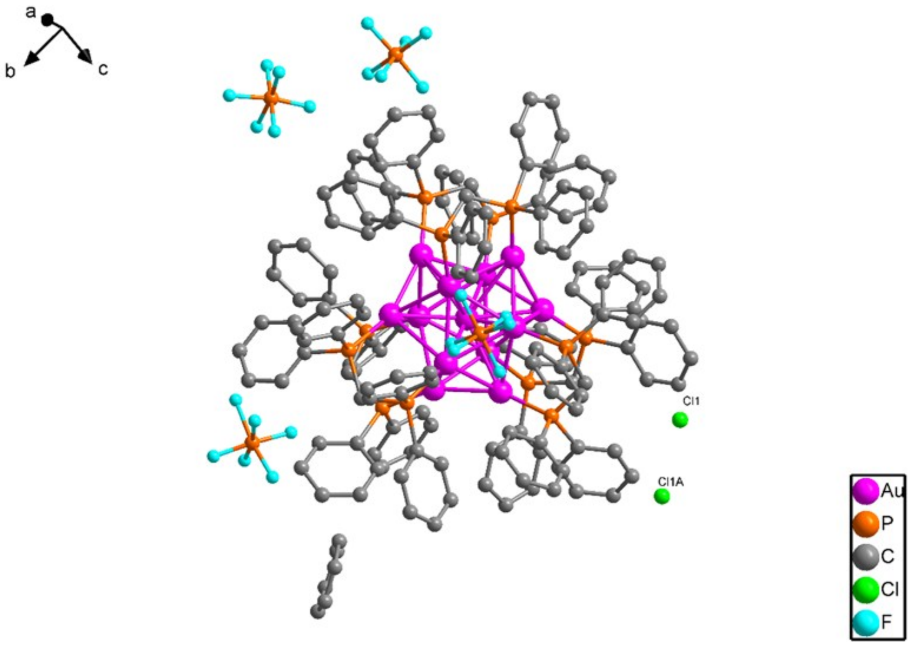

Figure S12: The unit cell of SD/Au1, SD/Au2 and SD/Au3 crystals. For clarity, H atoms and anions are omitted.

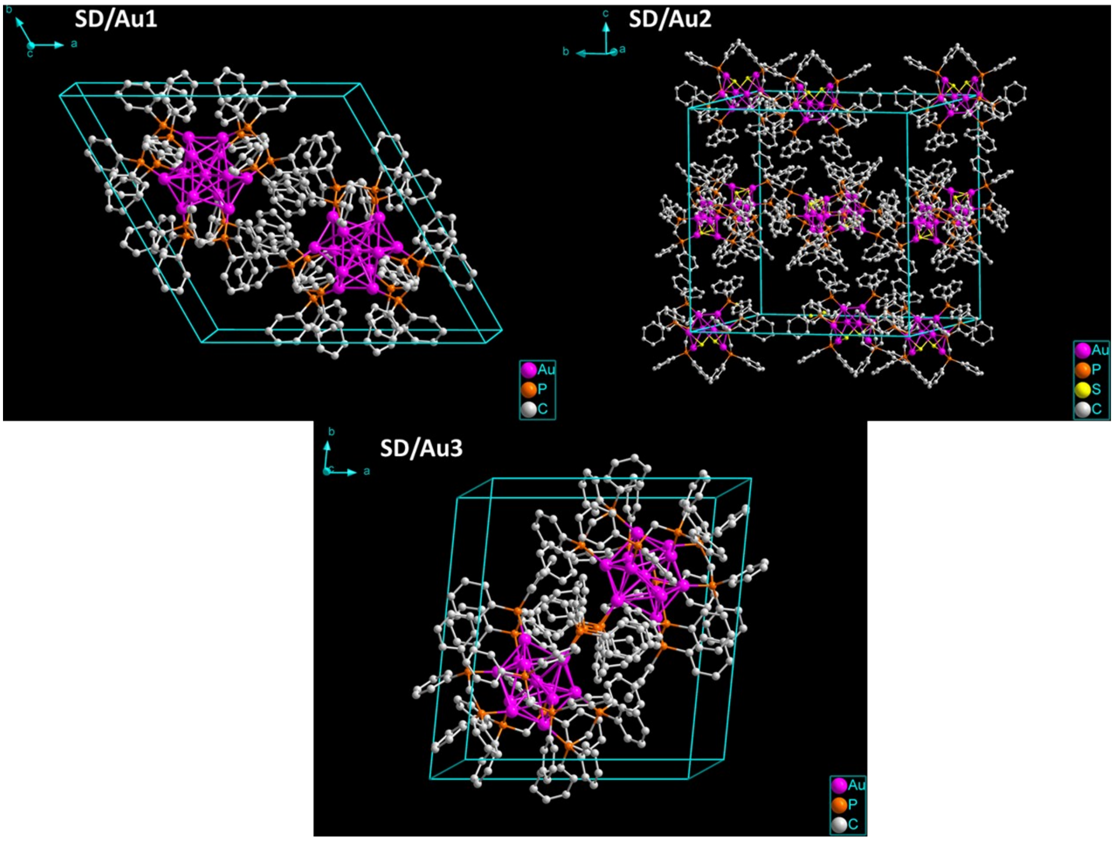

Figure S13: The powder X-ray diffraction patterns of SD/Au1·5Cl (a) and SD/Au2·2Cl (b).

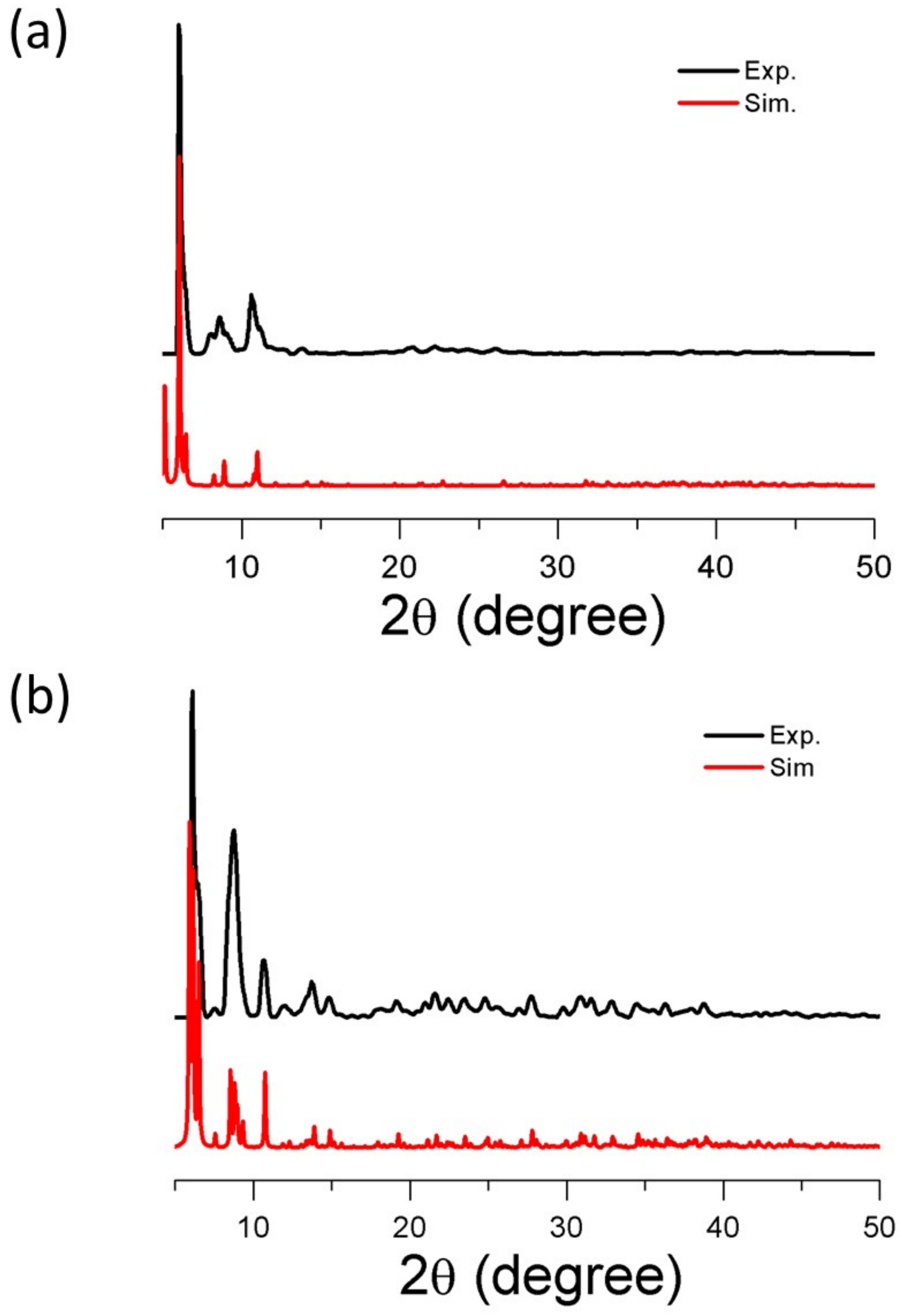

Table S7: Compared UV-vis spectra of previous Au_{13} (Mingos), Au_{13} (Konishi), and SD/Au1.

Author	Reference	Formula	Major Peak (nm)
Konishi	Nanoscale, 2012, 4, 4125–4129	$[Au_{13}(dppp)_5Cl_2]\cdot Cl$	340 and 430
		$[Au_{13}(dppb)_5Cl_2]\cdot Cl$	340 and 430
		$[Au_{13}(dpppe)_5Cl_2]\cdot Cl$	340 and 430
		$[Au_{13}(POct_3)_8Cl_4]\cdot Cl$	340 and 430
Mingos	J. Chem. Soc., Dalton. Trans. 1996, 491-500	$[Au_{13}(PMe_2Ph)_8Cl_4]\cdot(C_2B_9H_{12})$	345 and 430
This work		$[Au_{13}(dppm)_6]\cdot 5Cl$	440

Figure S14: The theoretical spectrum calculated for SD/Au1 where the phenyls in the dppm ligands are replaced by H ligands (a) for +5 charge state (b) for +3 charge state.

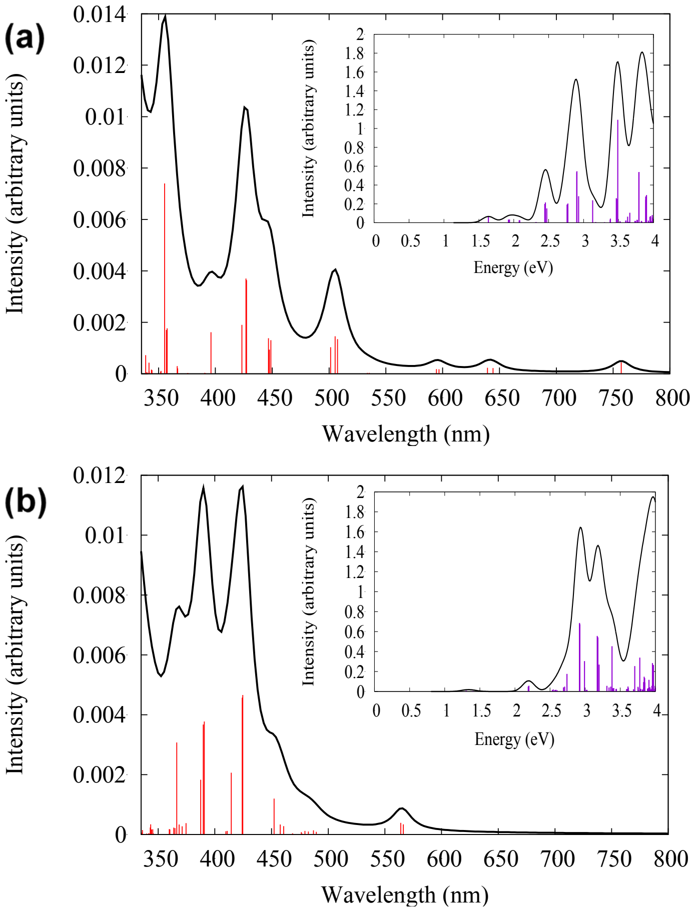

The +5 charge state spectrum shown here is for the case where we replaced the phenyl groups in the dppm ligands by hydrogens. Compared to the **SD/Au1** +5 charge state with full ligand spectrum shown in Figure 4 in the main text, the +5 charge state with H ligand spectrum shows similar peaks with a small shift in the peaks.

The computed optical absorption spectra for the two charge states demonstrates differences in the energy range of 450–800 nm. In **SD/Au1** with the +5 charge state, the spectrum shows peaks around 757, 639, 594 and 505 nm while the +3 charge state demonstrates only one peak around 566 nm. The +5 charge state also gives high intensity and higher energy peaks with strong oscillator strengths in the wavelengths around 447, 427, 396 and 357 nm. The +3 charge state gives similar peaks in the higher energy range at 457, 425, 391 and 374 nm.

Figure S15: The IR spectra of SD/Au1·5Cl (a), SD/Au2·2Cl (b) and SD/Au3·4PF_6·Cl·C_6H_6 (c).

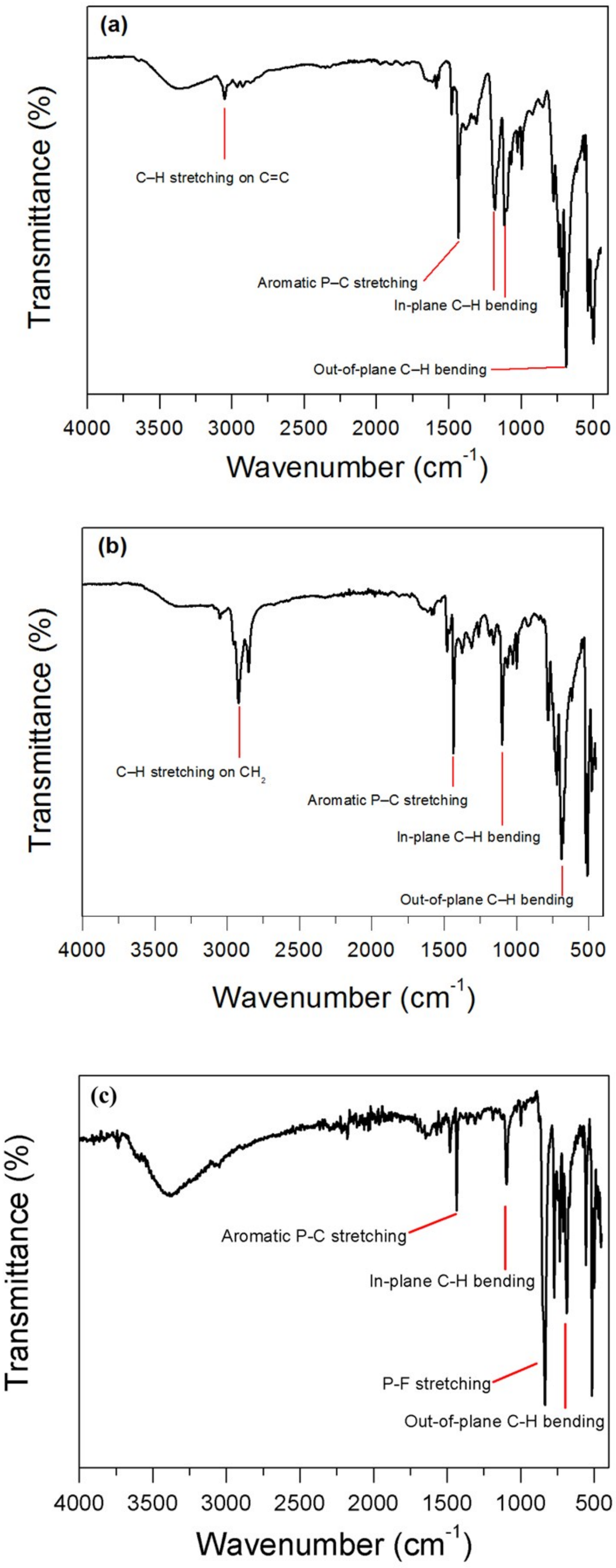

Supplement: Supplementary file 1 [file SC-009-C7SC03566G-s001.pdf]
